# Phages enhance both phytopathogen density control and rhizosphere microbiome suppressiveness

**DOI:** 10.1128/mbio.03016-23

**Published:** 2024-05-23

**Authors:** Xiaofang Wang, Shuo Wang, Mingcong Huang, Yilin He, Saisai Guo, Keming Yang, Ningqi Wang, Tianyu Sun, Hongwu Yang, Tianjie Yang, Yangchun Xu, Qirong Shen, Ville-Petri Friman, Zhong Wei

**Affiliations:** 1 Jiangsu provincial key lab for solid organic waste utilization, Key lab of organic-based fertilizers of China，Jiangsu Collaborative Innovation Center for Solid Organic Wastes, Educational Ministry Engineering Center of Resource-saving fertilizers, Nanjing Agricultural University, Nanjing, China; 2 College of Agro-grassland Science, Nanjing Agricultural University, Nanjing, Jiangsu, China; 3 China National Tobacco Corporation Hunan Company, Changsha, Hunan, China; 4 Department of Microbiology, University of Helsinki, Helsinki, Finland; University of Nebraska-Lincoln, Lincoln, Nebraska, USA

**Keywords:** bacterial wilt disease, phage biocontrol, rhizosphere microbiome, *Ralstonia solanacearum*, soil suppressiveness

## Abstract

Bacteriophages, viruses that specifically target plant pathogenic bacteria, have emerged as a promising alternative to traditional agrochemicals. However, it remains unclear how phages should be applied to achieve efficient pathogen biocontrol and to what extent their efficacy is shaped by indirect interactions with the resident microbiota. Here, we tested if the phage biocontrol efficacy of *Ralstonia solanacearum* phytopathogenic bacterium can be improved by increasing the phage cocktail application frequency and if the phage efficacy is affected by pathogen-suppressing bacteria already present in the rhizosphere. We find that increasing phage application frequency improves *R. solanacearum* density control, leading to a clear reduction in bacterial wilt disease in both greenhouse and field experiments with tomato. The high phage application frequency also increased the diversity of resident rhizosphere microbiota and enriched several bacterial taxa that were associated with the reduction in pathogen densities. Interestingly, these taxa often belonged to *Actinobacteria* known for antibiotics production and soil suppressiveness. To test if they could have had secondary effects on *R. solanacearum* biocontrol, we isolated Actinobacteria from *Nocardia* and *Streptomyces* genera and tested their suppressiveness to the pathogen *in vitro* and *in planta*. We found that these taxa could clearly inhibit *R. solanacearum* growth and constrain bacterial wilt disease, especially when combined with the phage cocktail. Together, our findings unravel an undiscovered benefit of phage therapy, where phages trigger a second line of defense by the pathogen-suppressing bacteria that already exist in resident microbial communities.

**IMPORTANCE:**

*Ralstonia solanacearum* is a highly destructive plant-pathogenic bacterium with the ability to cause bacterial wilt in several crucial crop plants. Given the limitations of conventional chemical control methods, the use of bacterial viruses (phages) has been explored as an alternative biological control strategy. In this study, we show that increasing the phage application frequency can improve the density control of *R. solanacearum*, leading to a significant reduction in bacterial wilt disease. Furthermore, we found that repeated phage application increased the diversity of rhizosphere microbiota and specifically enriched Actinobacterial taxa that showed synergistic pathogen suppression when combined with phages due to resource and interference competition. Together, our study unravels an undiscovered benefit of phages, where phages trigger a second line of defense by the pathogen-suppressing bacteria present in resident microbial communities. Phage therapies could, hence, potentially be tailored according to host microbiota composition to unlock the pre-existing benefits provided by resident microbiota.

## INTRODUCTION

Bacterial plant pathogens cause major economic losses to agricultural production due to lack of efficient control methods (1–3). While some disease control success has been achieved by using antibiotics and copper-based bactericides (4), the rapid evolution of multidrug-resistant pathogens and collateral damage to plant-beneficial microbiota have made their use infeasible (5, 6). As an alternative, pathogen-suppressing microbes have been suggested as potential biological control agents to limit damage caused by bacterial plant diseases (7). While several types of microbes have been tested (8), bacteriophages (viruses that infect bacteria; phages for short) have shown great promise in terms of being highly specific to target pathogens with different crops (9, 10). For example, previous work on *Ralstonia solanacearum* bacterium (the causal agent of bacterial wilt disease) has demonstrated that phage biocontrol is effective at reducing both pathogen densities and bacterial wilt disease symptoms (11, 12). However, the use of phages for disease protection has shown promising results, but the effectiveness of phage therapy can be hindered by various factors leading to incomplete protection. For example, phages might not be able to survive and find their host in heterogenous and often hostile soil environment (13–16), while pathogens could rapidly evolve resistant to phages, reducing the efficacy of phage therapeutics (12, 17). Furthermore, phage efficacy could depend on the surrounding microbiota and potential synergistic interactions with pathogen-suppressing bacterial taxa (12, 17). As a result, optimizing phage therapy might require considering both phage application approaches (e.g., combinations and timing) and potential synergies with the surrounding microbiota when pathogens reside and need to be treated in complex microbiomes.

Phages are often applied as multi-phage combinations to improve pathogen density control, increase phage cocktail infectivity range, and potentially constrain phage resistance evolution via differential receptor usage and trade-offs (12, 18–23). Furthermore, the timing, frequency, and dosage of phage applications can be optimized to improve phage efficacy (24, 25). For example, repeated application of phage cocktails has been shown to reduce pathogen loads and improve mouse survival in previous studies involving clinical bacterial pathogens (26, 27). Similar improvement in phage efficacy was obtained against plant pathogens when applied once or twice per week during the crop season (28–30). One likely reason for this is increased colonization and proliferation success of phages, which can lead to more frequent pathogen-phage encounter rates (31, 32). However, it remains less clear how phages affect and are affected by the surrounding resident microbiota during phage applications.

Plants are embedded in complex microbial communities that act as the first line of defense against invading pathogens (33, 34). While phage therapeutics often target the dominant pathogenic species (35), they could also indirectly shape the composition and functioning of rhizosphere microbiomes by freeing up niche space and triggering community reassembly when target pathogen densities are reduced (12, 33, 36–39). When used prophylactically, phages could prevent pathogens taking over the niche space from the other taxa (40), potentially stabilizing rhizosphere microbiomes. Such patterns were observed in a previous study, where *R. solanacearum*-specific phage cocktail buffered the resident microbiota against changes caused by the pathogen invasion (12). The application of phage cocktail has also been associated with changes in the proportion of taxa that showed facilitative or antagonistic pairwise interactions with the pathogen (12), and recently, healthy plants were associated with higher abundances of *R. solanacearum*-specific phages and a higher proportion of bacterial taxa that showed antagonism toward the pathogen (12, 38, 41). Together, these findings suggest that phage efficacy could be microbiome context dependent and potentially boosted or hindered by resident bacterial taxa.

Here, we specifically tested this by asking how phage application frequency affects the phage biocontrol efficacy of *R. solanacearum* in complex tomato rhizosphere microbiomes. *R. solanacearum* is a notorious phytopathogenic bacterium, which can infect several important crop species, resulting in serious economic losses globally (42, 43). Our previous work demonstrated that phage efficacy could be improved by applying phages as cocktails (12) or by combining a phage with a *Bacillus amyloliquefaciens* bacterium that suppresses *R. solanacearum* by producing antimicrobials (44). Here, we tested if the efficacy of phage therapy could be further improved by increasing the frequency of phage-cocktail application in tomato rhizosphere microbiomes. Our results show that increasing the application frequency improves the phage biocontrol efficacy through more efficient pathogen density control in both greenhouse and field experiments. No concomitant rise in phage resistance was observed. However, high phage application frequency was associated with increase in rhizosphere microbiome diversity and the relative abundance of pathogen-suppressing Actinobacteria. With further experiments, we causally show that the enriched Actinobacteria can synergistically suppress *R. solanacearum* when combined with the phage cocktail. Together, our findings reveal an undiscovered benefit of phage therapy, where phage application can activate pathogen suppression of the pre-existing resident microbiota.

## MATERIALS AND METHODS

### Bacterial strains and phage cocktail design

*Ralstonia solanacearum* strain QL-Rs1115 (45) was used as a model pathogenic strain and grown at 30°C in NB medium (10 g glucose, 5 g tryptone, 3 g beef extract, and 0.5 g yeast extract/L) for 24 h with shaking (170 rpm) before all the experiments. Selective M-SMSA agar plates were used to calculate *R. solanacearum* densities in the rhizosphere (46). Phage cocktail with four lytic podophages (NJ-P3, NB-P21, NC-P34, and NN-P42, Table S1; Fig. S1) that had been isolated from four geographically distant tomato fields in China was used in all experiments (NJ: Nanjing, NB: Ningbo, NC: Nanchang, and NN: Nanning [12]). Phage genome sizes were 42,528 base pairs for NJ-P3, 41,194 bps for NB-P21, 41,943 bps for NC-P34, and 42,278 bps for NN-P42. Average GC contents were 62.26% for NJ-P3, 62.22% for NB-P21, 61.99% for NC-P34, and 62.10% for NN-P42. All four isolated phages were closely related with each other (>99.93% genetic similarity) and belonged to *Peduviridae* family based on the ICTV classification (Fig. S1). All phage types were distantly related with known lysogenic phages publicly available in NCBI and showed clear lysis on the used *R. solanacearum* strain on double-layer agar plates, indicative of the lytic nature. To prepare phage stocks, each phage was grown individually with the stock QL-Rs1115 in NB medium (12) for 24 h as described above with the addition of centrifugation (5 min at 10,000 × *g*) and filtration (0.22 µm) steps at the end to isolate and purify phages from bacteria. Phage titers were adjusted to 10^7^ phage particles per milliliter, and phage stocks were stored at 4°C. Semi-selective GS media (Gause’s synthetic agar no. 1: soluble starch 20 g L^−1^, KNO_3_ 1 g L^−1^, NaCl 0.5 g L^−1^, K_2_HPO_4_ 0.5 g L^−1^, MgSO_4_·7H_2_O 0.5 g L^−1^, FeSO_4_·7H_2_O 0.01 g L^−1^, and agar 20 g L^−1^) were used to isolate *Actinomycetes* strains from the soil (47), and the International *Streptomyces* Project (ISP) 3 medium (containing 20 g L^−1^ of oatmeal; 1 mg L^−1^ of FeSO_4_·7H_2_O, MnCl_2_·H_2_O and ZnSO_4_·7H_2_O each, and 18 g L^−1^ of agar; the final pH adjusted to 7.2) was used to purify and culture *Actinomycetes* strains (48). To prepare the *Actinomycetes* stocks for the greenhouse experiment, the *Streptomyces* spores growing in ISP3 agar medium for 7 days at 30°C were washed with sterile water, and spore suspensions with 10^8^ mL^−1^ concentration were prepared by hemocytometer counting plate method.

### Greenhouse and field experiments

Surface-sterilized tomato seeds (*Lycopersicon esculentum*, cultivar “Micro-Tom”) were germinated on water-agar plates for 3 days before sowing into 54-well trays filled with growth substrate (50 g substrate per well; commercially available from Jiangsu-Xingnong Substrate Technology Co., Ltd). At the three-leaf stage, plants were transplanted into 6-well trays with scaled-down “rhizobox” systems (41) to quantify the pathogen abundance dynamics in the rhizosphere. This system allows repeated, non-destructive sampling of a subset of rhizosphere soil along with removable nylon bags without damaging the root system. Each well contained 200 g of non-sterilized natural topsoil (50 g per well; collected from a tomato field in Qilin, 118°57ʹE, 32°03ʹN) (12) and six separate nylon mesh bags outside the cells that could be removed individually during sampling. The type of the Qilin soil was yellow-brown soil, which is typically used for growing vegetables (12). It contained 24.0 g kg^−1^ of organic matter, 1.7 g kg^−1^ of total nitrogen, 173.1 mg kg^−1^ of available phosphorus, and 178 mg kg^−1^ of available potassium and had a pH of 5.8. No pesticides were used during the experiment, and a standard chemical fertilization was applied. One week later, *R. solanacearum* stock strain was inoculated to plant roots at a final concentration of 10^6^ colony-forming units (CFU) g^−1^ soil [mixed in 10 mL of SM buffer including 5.8 g L^−1^ NaCl, 2.0 g L^−1^ MgSO_4_·7H_2_O, 50.0 mL L^−1^ of 1 M Tris-HCl (pH 7.5), and 5.0 mL L^−1^ 2% (wt/vol) of gelatin]. The phage cocktail (all phages in equal proportions at a final density of approximately 10^5^ plaque-forming units [PFU] g^−1^ soil) was applied once (day 2), two times (days 2 and 9), or three times (days 2, 9, and 16) after pathogen inoculation, while the control treatment included only pathogen inoculation without phages. A total of 24 tomato plants were used, resulting in 4 replicates per treatment (*N* = 4), where every replicate consisted of 6 individual plants. Plants were grown for 25 days in a greenhouse with a natural temperature variation ranging between 25°C and 35°C, and seedling trays were randomly rearranged every 3 days. Disease index was recorded every day post pathogen infection (dpi) based on a scale ranging from 0 to 4, where 0 denotes zero disease incidence, 1 denotes 1%–25% disease incidence, 2 denotes 26%–50% disease incidence, 3 denotes 51%–75% disease incidence, and 4 denotes 76%–100% disease incidence (49). Rhizosphere soil samples (2.0 g) were collected from two randomly chosen healthy plants (one bag per plant) from replicate trays 1 day before and 1 day after phage-cocktail application. With each replicate, two plant samples were homogenized thoroughly and pooled into one composite sample per sampling time point to increase the representativeness of the sampling. Final rhizosphere soil samples were collected at the end of the experiment at day 25. All rhizosphere soil samples were used to calculate pathogen and phage population densities, and the final samples were stored at −80°C for DNA extraction and determination of bacterial community composition (described in more detail later).

The field experiment was conducted at Baima, Nanjing, China (116°23*'*29*"*E, 39°54*'*19*"*N) where we tested the effect of phage cocktail application frequency (once vs three times) on the biocontrol efficacy relative to a no-phage control treatment. The experiment was conducted between October and November 2022 with 25°C maximum and 10°C minimum daily temperatures (the average temperature during the experiment was 20°C). Due to the outdoor field experiment conducted in November, we did not employ additional temperature control measures. The ambient temperature fluctuated considerably, occasionally reaching approximately 10℃. However, for the majority of the time, the temperature remained above 20°C, facilitating normal tomato growth. The type of soil was red soil collected from Guangxi, which is typically used for growing vegetables in this area. It contained 15.4 g kg^−1^ of organic matter, 1.4 g kg^−1^ of total nitrogen, 11.7 mg kg^−1^ of available phosphorus, and 113.0 mg kg^−1^ of available potassium and had a pH of 4.5. The soil was naturally infected by *R. solanacearum* (>10^6^ CFU g^−1^ soil), resulting in typically high wilt disease incidence (>80% tomato plants infected). The tomato seedlings (cultivar Hongaisheng) were first grown in nursery trays for 30 days before transplantation to larger individual pots. Each treatment contained five replicate blocks in random block design. Within each block, nine plants were spaced out evenly approximately 30 cm apart from each other in three rows. After 7 days from transplantation, the same four-phage cocktail, which was used in the greenhouse experiment (~10^8^ total PFUs per plant), was applied to the roots of phage treatment tomato plants (mixed in 100 mL M9 buffer), while no-phage control treatment was treated with the same volume of M9 buffer without phages. The M9 buffer was also applied onto plants treated with phages only once during the weeks 2 and 3 (days 14 and 21), while plant assigned to three-application treatment received the same phage cocktail during the weeks 2 and 3. The severity of bacterial wilt disease was recorded 40 days after inoculation as a mean of disease index of each plant using a scale ranging from 0 to 4 described earlier.

### Quantification of *R. solanacearum* and phage densities

To quantify *R. solanacearum* densities during the greenhouse experiment, 1 g of rhizosphere soil was mixed with 9 mL water, vortexed thoroughly, and supernatant serially diluted on M-SMSA medium agar plates (46). Colony-forming units were counted after incubation at 30°C for 2 days. The same soil supernatants were centrifuged and filtered (0.22 µm) for phage densities detection using a spotting assay on soft double-agar overlays of *R. solanacearum* stock strain (44). After 24 h of growth at 30°C, phage densities were calculated by counting plaque-forming units per milliliter of phage suspensions.

### Measuring *R. solanacearum* resistance evolution during greenhouse experiment

For the greenhouse experiment, 3 *R. solanacearum* colonies from each treatment replicate soil samples were isolated after 8 and 15 days from pathogen inoculation, resulting 12 colonies for each treatment at 1 time point, and used for phage resistance measurements (a total of 24 colonies per treatment). The resistance of each colony was scored against each ancestral phage included in the cocktail using the cross-streaking assay (23, 50, 51), which tests if bacterial growth is inhibited when streaked across a “line” of dried phage (40 µL) on NA plate (12). Strains were scored as sensitive if there was detectable inhibition of growth by the phage compared to the control treatment (bacteria streaked on plates without phages).

### Determining changes in bacterial community composition using 16S rRNA amplicon sequencing

Changes in bacterial community composition were quantified at the end of the greenhouse experiment for all treatment replicates (two plants combined as a composite sample per replicate). The DNA was extracted using a Power Soil DNA Isolation kit (Mo Bio Laboratories) following the manufacturer’s protocol. The DNA concentration and quality were determined by using a NanoDrop 1000 spectrophotometer (Thermo Scientific) and electrophoresis for DNA shearing. 16S rRNA amplicon sequencing was used to determine bacterial community composition and diversity. The V4 region of the bacterial 16S rRNA genes was amplified using a primer set 563F and 802R (52). The amplicon library was paired-end sequenced (2 × 250) on an Illumina MiSeq platform (Shanghai BIOZERON Co., Ltd) by following standard protocols (53). Sequence reads were dereplicated and processed using the DADA2 algorithm in QIIME 2 (54). The trimming and filtering were performed on paired reads with a maximum of two expected errors per read (maxEE = 2). After merging the paired reads and chimera filtering, the phylogenetic affiliation of each 16S rRNA gene sequence (herein called ASVs) was analyzed using UCLUST algorithm (http://www.drive5.com/usearch/manual/uclust_algo.html) against the silva (SSU138.1) 16S rRNA database with confidence threshold level of 80% (55). Only sequences with total abundances greater than 5 were retained in the data set for further analysis. The raw reads were deposited into the NCBI Sequence Read Archive database (accession number: PRJNA819579).

### Culture-dependent assessment of *R. solanacearum* suppression by Actinobacteria

To validate the patterns observed in sequencing data, we isolated *Actinomyces* strains from the healthy rhizosphere soil samples at the end of the greenhouse experiment and determined their antimicrobial activity against *R. solanacearum* stock strain. All replicate samples belonging to any of the phage treatments were pooled and homogenized after 5 g of soil samples was mixed with 45 mL of sterilized water, vortexed thoroughly, and supernatants serially diluted on GS agar media supplemented with cycloheximide (50 mg L^−1^) and nalidixic acid (20 mg L^−1^) (47). After 20 days of incubation at 28°C, 10 candidate *Actinomyces* isolates were randomly isolated from the plates based on colony morphology and further purified by culturing on ISP3 agar plates (48) and stored in glycerol suspensions (30%, vol/vol) at −80°C. These 10 strains were characterized taxonomically based on their 16S rRNA gene sequences as described earlier.

The antimicrobial activity of 10 isolated *Actinomyces* strains was estimated by testing the inhibitory effect of their cell-free filtrates against the *R. solanacearum* stock strain (56). To this end, 10^7^ mL^−1^ of each *Actinomyces* strain was inoculated into 700 µL of liquid NB medium and cultured at 30°C for 5 days, after all bacterial cultures were filter sterilized to remove living cells (0.22 µm filter). Subsequently, 20 µL of sterile supernatant from each *Actinomyces* strain culture and 2 µL overnight culture of *R. solanacearum* (adjusted to OD_600_ = 0.5 after 12 h of growth at 30°C with shaking) were added into 180 µL of fresh NB medium. Control treatments were inoculated with equal volume of sterile water instead of the bacterial supernatant. Each treatment was replicated for three times. All bacteria-supernatant cultures were grown for 24 h at 30°C with shaking (170 rpm) before measuring OD_600_. The inhibition effect was calculated as the difference in bacterial growth in the control (OD_600c_) and supernatant (OD_600s_) treatments using the following formula: inhibition effect = (OD_600c_ – OD_600s_)/OD_600c_.

One strain of *Streptomyces* and one strain of *Nocardia* were randomly selected for antagonist activity against *R. solanacearum* QL-Rs1115 using a spot-spraying method on NA agar plates (45). Briefly, Actinobacteria isolates were spotted with toothpicks on Gauze’s no.1 plates and cultivated for 7 days at 30°C. Actinobacteria blocks with diameter of 6 mm were removed using a sterile punch and placed on a new NA plate, which was subsequently inoculated with *R. solanacearum* QL-Rs1115 stock strain suspension (10^8^ CFU mL^−1^), which was sprayed once (∼0.2 mL) onto the plates. The plates were then incubated at 30°C for 24 h, and the diameter of the *R. solanacearum* inhibition zones around Actinobacteria blocks was used as a measure of antimicrobial activity.

### Validating the suppressiveness of isolated *Actinomyces* strains in the absence and presence of phage cocktail

Based on the phylogenetic dissimilarity and antimicrobial activity, two isolated *Streptomyces* and *Nocardia* strains were used to test their suppressiveness alone and in combination with four-phage cocktail in a separate greenhouse experiment. The tomato plants were prepared the same way as described earlier, and six treatments were included in the experiment (Control, pathogen only; P, phage cocktail; N, *Nocardioides* alone; S, *Streptomyces* alone; PN, phage cocktail and *Nocardioide*s; PS, phage cocktail and *Streptomyces*). Each treatment consisted of 24 tomato plants that were blocked in 4 replicates of 6 plants. After 7 days from transplanting to six-cell trays, a subset of plants was inoculated with *Nocardioide*s or *Streptomyces* strains with a final concentration of 10^5^ CFU g^−1^ soil as per the factorial experimental design. While such densities might not occur naturally in soil, high bacterial densities were chosen to increase the likelihood of detecting potential synergistic effects. Moreover, such densities are still achievable in bioreactor growth conditions, making densities feasible for bioinoculation in practice. After 5 days of inoculation of *Actinomyces* strains, *R. solanacearum* stock strain was inoculated to all plants (except for no-pathogen negative control) at a final concentration of 10^6^ CFU g^−1^ soil. The phage cocktail was applied last after 2 days of inoculating *R. solanacearum*, with a final density of approximately 10^6^ PFU g^−1^ soil. The severity of bacterial wilt disease was recorded as described earlier.

### Statistical analyses

Pathogen and phage density data were log 10 transformed to fulfill the parametric model assumptions. The time-dependent data were analyzed using repeated measures ANOVA using replicates as subjects and treatment and time as explanatory variables. The relative pathogen abundance was calculated as the proportion of *R. solanacearum* ASVs of all bacterial ASVs. Species richness and Simpson diversity indexes were calculated using the “diversity” function in R packages vegan. All *R. solanacearum* ASVs were removed before diversity analysis to concentrate on changes in rhizosphere bacterial community composition. To explore how changes in bacterial phylum abundances were associated with pathogen and phage density changes in the rhizosphere, Spearman rank correlation was used. Principal component analysis was applied to compare the differences in community composition between treatments using the PERMANOVA test with R packages vegan (57); *R. solanacearum* ASVs were included in this analysis.

To identify sensitive taxa (biomarkers) that showed abundance changes in response to phage cocktail application frequency, we conducted correlation-based indicator analysis with the multipatt function in the R package indispecies (58). To this end, we first calculated the point-biserial general correlation coefficient (*r.g*.) for ASVs that were positively associated with phage-cocktail application frequency, and the strength and statistical significance of the relationship between species occurrence and treatments were determined after 999 permutations with cut-off line of *P* < 0.05 (59).

The most common genera whose appearance frequency was ≥50% were also included in the co-occurrence network analysis. Package “Hmisc*”* (60) was used to calculate pairwise Spearman correlation matrix, and package “fdrtool” (61) was used to adjust the corresponding *P*-values. Correlations with pairwise Spearman absolute *r*-values >0.90 and adjusted *P*-values <0.05 were only kept in the final co-occurrence network. Function “cluster_fast_greedy*”* from package “igraph*”* (62) was used to cluster co-occurrence network modules according to Newman algorithm to identify groups of taxa that responded similarly to phage cocktail application frequency. To identify key predictor taxa, random forest (RF) approach by package “randomForest*”* with 500 of trees was used (63).

One-way analysis of variance and Kruskal-Wallis rank sum tests were used to compare pairwise differences between treatment groups, depending on the data distribution and homogeneity of variance. Duncan’s multiple range test was used to compared pairwise differences between any two groups. All analyses were conducted using SPSS v16.0 and R version 4.1.1 (64), and *P*-values <0.05 were considered statistically significant.

## RESULTS

### Repeated phage cocktail application improves bacterial wilt disease biocontrol efficacy

**Fig 1:**
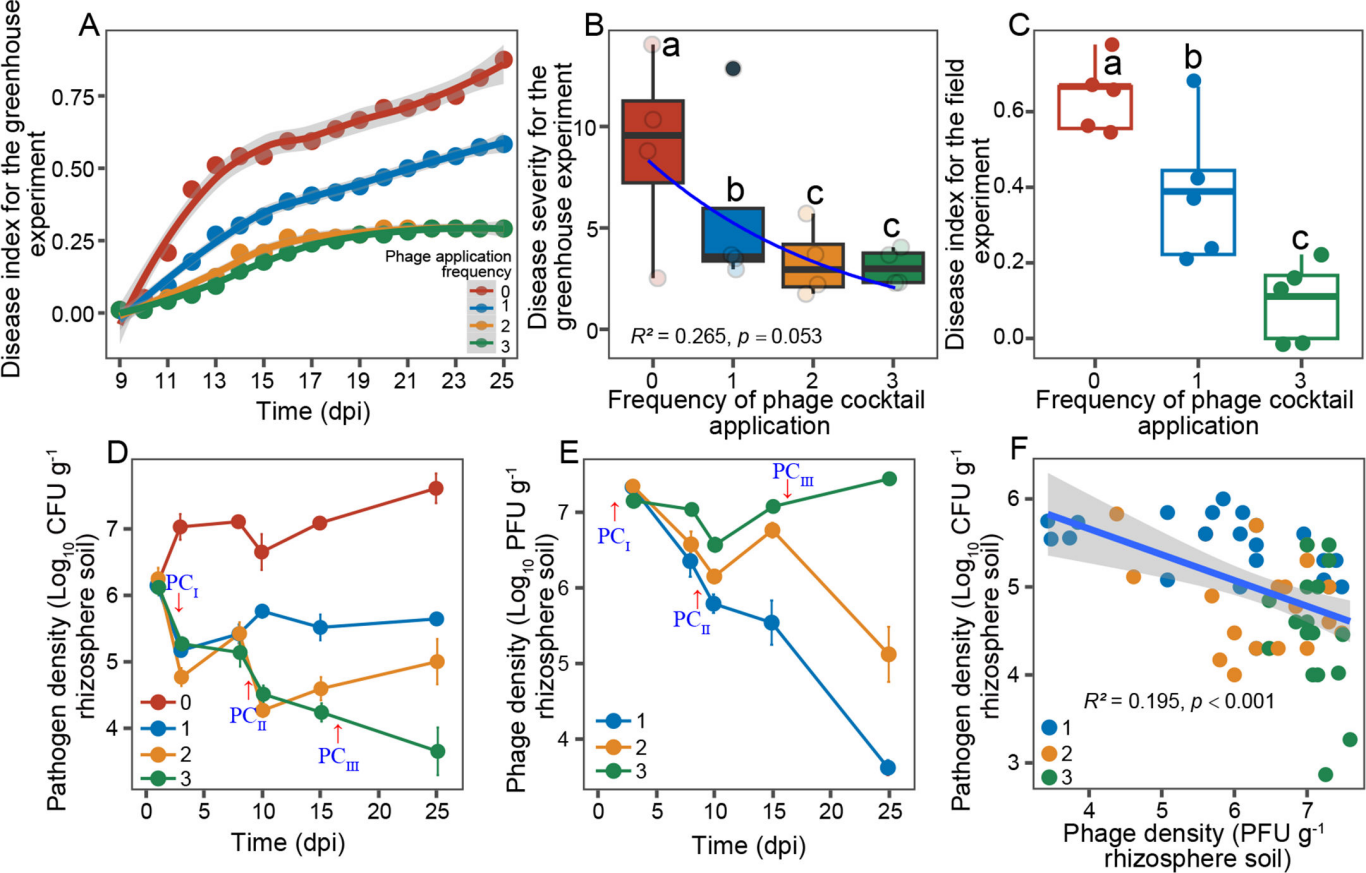
The effect of repeated phage cocktail application on bacterial wilt disease dynamics and pathogen and phage densities in greenhouse and field experiments. Panels **A** and **B** show bacterial wilt disease dynamics and average disease severity between different phage application frequency treatments at the end of the greenhouse experiment, respectively (*N* = 4). Disease severity was calculated as an area under the disease index curve. Panel **C** shows differences in bacterial wilt disease incidence (disease index) between different phage application treatments at the end of the field experiment (*N* = 5). Lowercase letters in panels **B** and **C** denote statistically significant differences between phage treatments (Duncan’s multiple range test). Panels **D** and **E** show pathogen and phage density dynamics in the rhizosphere between different phage treatments during the greenhouse experiment; red arrows indicate the phage cocktail application time points (*N* = 4). Panel **F** shows negative correlation between pathogen and phage densities at the final sampling time point of the greenhouse experiment. The *x*-axes in panels **A**, **D**, and **E** represent the days post inoculation of *R. solanacearum*. The line in panel **B** is fitted with nonlinear regression analysis (equation: sigmoidal, sigmoid, 3 parameter). The line in panel **F** shows a fitted linear regression and SEM as shaded area around the mean. The red color in the panels denotes the control treatment, while blue, yellow, and green lines represent the phage applied once, two times, or three times, respectively. Panel **C** illustrates the results of the field experiment; yellow line is absent as treatment where phage was applied for two times was not included in this experiment. Panels **E** and **F** depict the outcomes of the greenhouse experiments, and since no phage was detected in the control treatment, red lines are not displayed in panels **E** and **F**.

We first used a greenhouse experiment to test if bacterial wilt biocontrol efficacy could be improved by increasing the phage cocktail application frequency. Bacterial wilt disease index rapidly increased after 8-day initial lag phase in the pathogen-only control treatment, resulting in relatively highest disease index score by the end of the greenhouse experiment (*F*_3,15_ = 21.137, *P* < 0.001, Fig. 1A). The phage cocktail application considerably reduced the bacterial wilt disease progression. While the single phage cocktail application reduced the disease incidence by 33% (*F*_1,7_ = 12.783, *P* = 0.012), applying phage cocktail two or three times led to a much greater reduction (average of 67%) in bacterial wilt disease compared to the pathogen-only control treatment (*F*_1,11_ = 60.775, *P* < 0.001, Fig. 1B). Similar patterns were also observed in the field experiment. While applying phages only once led to statistically significant reduction in the bacterial wilt disease compared to the control treatment (40%), this effect was greater (84%) when phages were applied three times (*F*_2,14_ = 21.212, *P* < 0.001, Fig. 1C).

We next explored how phage cocktail application frequency affected *R. solanacearum* and phage densities during the greenhouse experiment. Pathogen population densities clearly increased from the initial inoculation of 10^6^ CFU/g of soil in the control treatment, while the application of phage cocktail significantly constrained the *R. solanacearum* growth in the rhizosphere (treatment: *F*_3,69_ = 186.66, *P* < 0.001; time: *F*_5,69_ = 14.14, *P* < 0.001, Fig. 1D). While some recovery in *R. solanacearum* densities was observed when phages were applied once or twice, consistent reduction with four orders of magnitude relative to the control treatment was observed when phages were applied three times (*F*_1,7_ = 87.323, *P* < 0.001). Inverse pattern was observed with phage density data. While phage densities decreased from the initial inoculation of 10^7^ to less than 10^4^ PFU/g of soil when applied only once or twice, no reduction in phage densities was observed when phages were applied three times (*F*_2,11_ = 75.371, *P* < 0.001, Fig. 1E). Overall, pathogen densities correlated negatively with phage densities at the final sampling time point (*R*^2^ = 0.195, *P* < 0.001, Fig. 1F).

We also tested whether rapid evolution of phage resistance took place during the greenhouse experiment (12) by testing the susceptibility of *R. solanacearum* colonies isolated from all the treatment replicates before the second and third phage application time point against all four ancestral phages. We found that all isolated *R. solanacearum* colonies were susceptible to all ancestral phages (Fig. S2), suggesting that the observed pathogen density differences between phage treatments were unlikely affected by phage resistance evolution. Together, these findings demonstrate that *R. solanacearum* biocontrol can be improved by applying phages repeatedly during the tomato growth cycle.

### Repeated phage application had clear effects on the diversity and composition of tomato rhizosphere microbiota during the greenhouse experiment

**Fig 2:**
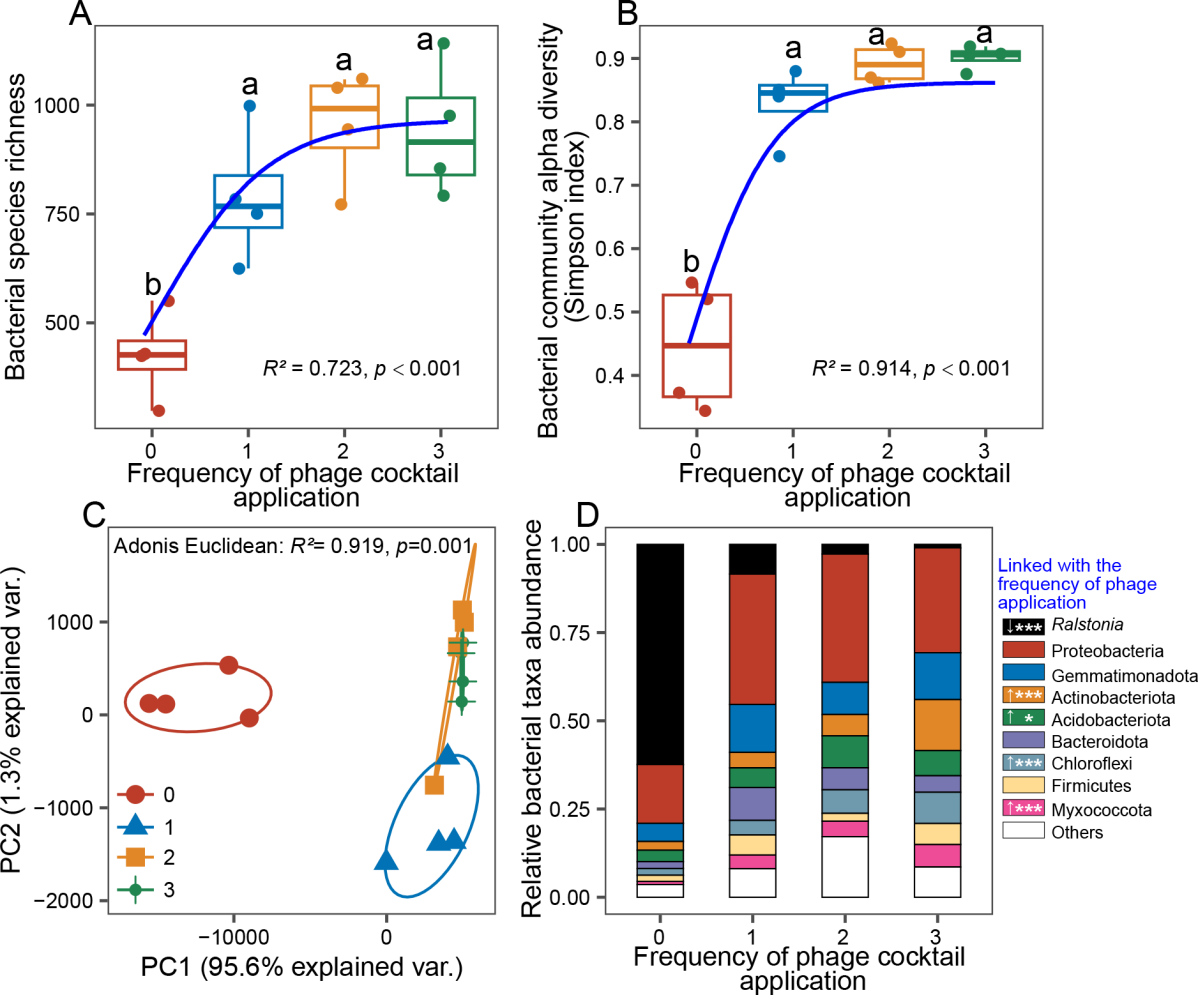
The effect of phage application frequency on the diversity and composition of rhizosphere bacterial communities in greenhouse experiment. Panels **A** and **B** show bacterial taxa richness and Simpson diversity, respectively (*N* = 4), and lowercase letters denote statistically significant differences between treatments (Duncan’s multiple range test). Panel **C** shows bacterial community structure visualized based on principal component analysis (*N* = 4). Panel **D** shows the effect of phage application frequency on the relative abundance of the top 8 most abundant bacterial phyla (*N* = 4); the key on the right shows the significance and direction of changes for each phylum and the pathogen (upward and downward arrows denote increases and decreases in abundances, respectively, and the stars denote significant changes along with phage application frequency treatment). The red color in panels **A**, **B**, and **C** denotes the control treatment, while blue, yellow, and green lines represent the phage applied once, two times, or three times, respectively. Lines in panels **A** and **B** are fitted with nonlinear regression analysis (equation: sigmoidal, sigmoid, 3 parameter).

We next compared the effect of phage application frequency on the bacterial community diversity and composition at the final sampling time point of the greenhouse experiment. Overall, the phage application increased both bacterial community richness and diversity compared to control treatment (ASV richness: *F*_3,15_ = 12.899, *P* < 0.001; Simpson diversity: *F*_3,15_ = 49.869, *P* < 0.001, Fig. 2A and B), and phage effects on the diversity were the strongest when phages were applied two or three times (*F*_2,15_ = 80.651, *P* < 0.001, Fig. 2B). As a result, increasing the phage cocktail application frequency correlated positively with both bacterial community richness and diversity across all treatments (ASV richness: *R*^2^ = 0.723, *P* < 0.001; Simpson diversity: *R*^2^ = 0.914, *P* < 0.001, Fig. 2A and B). Similarly, phage application changed the bacterial community composition (Adonis test: *R*^2^ = 0.517, *P* = 0.001), and this effect was magnified with increasing phage application frequency, being especially clear along the PC1, which explained 95.1% of the total variation in bacterial community composition (Adonis test: *R*^2^ = 0.320, *P* = 0.015, Fig. 2C). In-line with the CFU data, the relative abundance of *R. solanacearum* ASVs decreased along with phage application frequency (*F*_3,15_ = 73.185, *P* < 0.001, Fig. 2D). Interestingly, the reduction in *R. solanacearum* relative abundances correlated positively with the relative abundances of Gemmatimonadota (*R*^2^ = 0.4, *P* = 0.02), Actinobacteria (*R*^2^ = 0.6, *P* < 0.001), Acidobacteriota (*R*^2^ = 0.4, *P* = 0.008), Chloroflexi (*R*^2^ = 0.5, *P* = 0.002), and Myxococcota (*R*^2^ = 0.8, *P* < 0.001) (Fig. S3). Moreover, increase in the relative abundances of Gemmatimonadota (*R*^2^ = 0.3, *P* = 0.04), Actinobacteria (*R*^2^ = 0.7, *P* = 2e-05), Acidobacteriota (*R*^2^ = 0.4, *P* = 0.008), Chloroflexi (*R*^2^ = 0.7, *P* < 0.001), and Myxococcota (*R*^2^ = 0.8, *P* < 0.001) were positively correlated with phage densities in the rhizosphere (Fig. 2D). Together, these results suggest that repeated phage cocktail application altered the diversity and composition of rhizosphere microbiota, leading to clear enrichment of certain bacterial taxa.

### Repeated phage application enriches specific bacterial co-occurrence modules in the rhizosphere during the greenhouse experiment

To understand if certain bacteria responded similarly to phage application frequency, we conducted an indicator species and network module analysis at the bacterial genus level for the greenhouse data. We found that a total of 48 bacterial genera could be considered as indicator taxa, which most often belonged to Proteobacteria (35.42%) and Actinobacteria (18.75%). These indicator taxa were grouped into “specific” if they were only present in one phage treatment and “core” if they were present in more than two phage application treatments. Interestingly, almost half of the identified indicator taxa (21 out of 48) were specific to the treatment where phages were applied for three times during the greenhouse experiment (Table S2).

**Fig 3:**
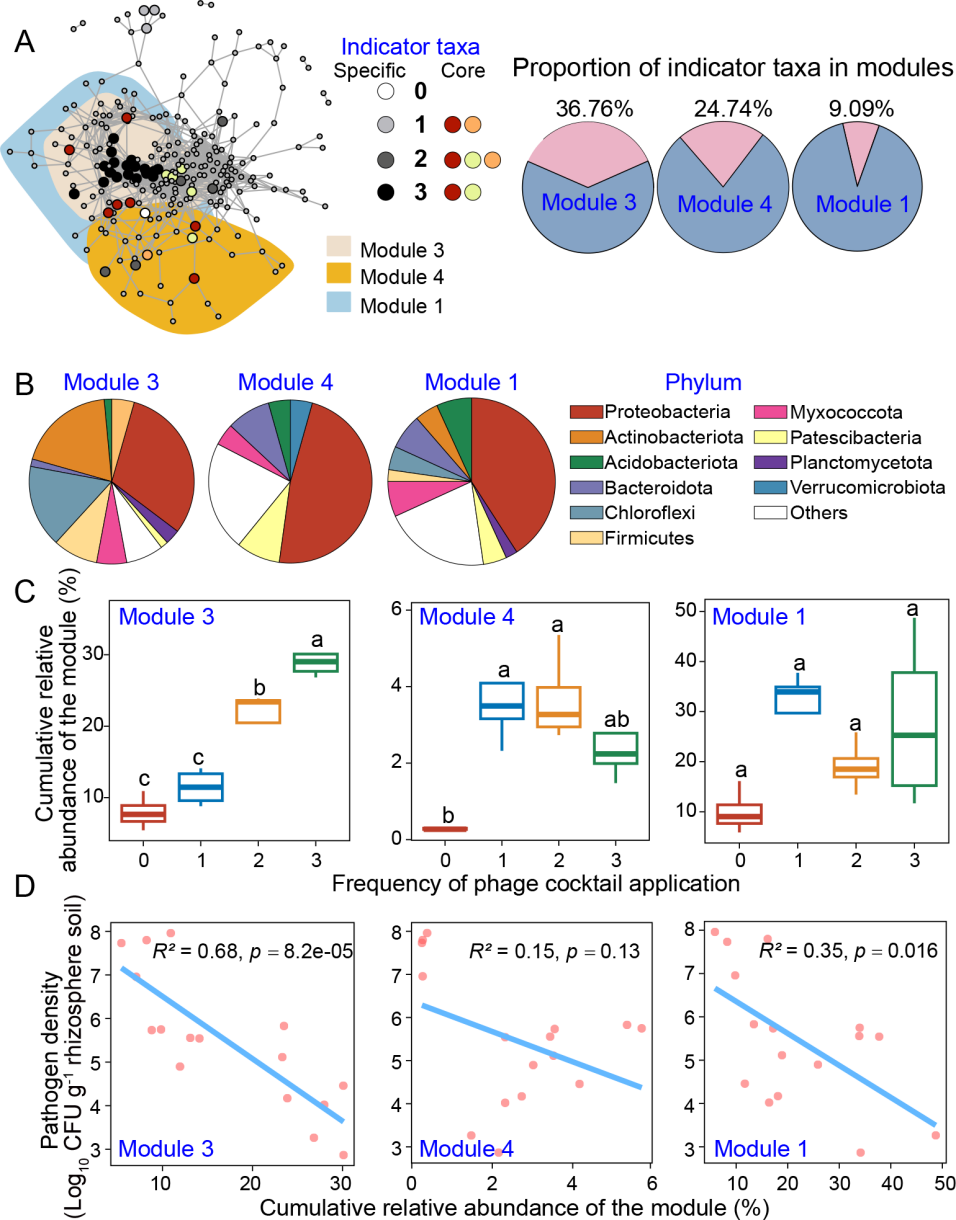
The co-occurrence of indicator bacterial taxa associated with phage application frequency in greenhouse experiment. Panel **A** shows the bacterial co-occurrence network (*R* > 0.75, *P* < 0.05) across the whole data set. Shaded colored areas represent modules that contain more than four indicator taxa. The node colors denote if the indicator taxa were considered as “specific” to a given phage application frequency treatment (grayscale) or “core” where indicator taxa were found across several or all phage treatments (colors). Pie charts on the left show the proportion of indicator taxa belonging to each module on pink. Panel **B** shows the taxonomic composition of modules 3, 4, and 1 (from top to bottom, respectively). Panels **C** and **D** show the cumulative relative abundances of indicator modules in different phage treatments and significant correlations with pathogen densities in the rhizosphere (lines show fitted linear regression). The red color in panel **C** denotes the control treatment, while blue, yellow, and green lines represent the phage applied once, two times, or three times, respectively. *N* = 4 for each treatment.

We next constructed co-occurrence networks based on all treatments to identify bacterial taxa that responded similarly to phage application frequency (Fig. 3A). A total of 13 modules could be identified from the co-occurrence network (Table S3), and 3 of these modules contained more than 4 indicator taxa. Specifically, module 3 contained 25 indicator taxa (36.7% of all taxa), which were dominated by Actinobacteria (32%) and Chloroflexi (20%). These taxa were mostly specific to treatment where phages were applied for three times. Modules 4 and 1 contained five (21.7% of all taxa) and four (9% of all taxa) indicator taxa, which mainly belonged to Proteobacteria (Fig. 3B). These indicator taxa were core to all phage treatments, and their abundances correlated positively with the phage application frequency. To find out how the relative module abundances responded to phage application frequency, we compared their cumulative abundance changes between phage treatments. While phage presence increased the module 4 (*H* = 10.147, *P* = 0.017) and module 1 abundances (*H* = 8.051, *P* = 0.045), the effect of phage application frequency was non-significant (all pairwise comparisons, *P* > 0.05). In contrast, module 3 abundances responded significantly with the phage application frequency (*H* = 12.794, *P* = 0.005, Fig. 3C), showing the highest relative cumulative abundances when the phage was applied for three times. Correlation analysis further revealed that the abundances of module 3 (*R*^2^ = 0.68, *P* = 8.2e−05) and module 1 (*R*^2^ = 0.35, *P* = 0.016) were negatively associated with pathogen densities in the rhizosphere (Fig. 3D). Together, these results suggest that co-occurring Actinobacteria and Chloroflexi that dominated module 3 responded positively to the increase in phage application frequency and the reduction in pathogen densities.

### *Nocardioides* and *Streptomyces* bacteria are potential pathogen-suppressing taxa associated with pathogen density reduction during the greenhouse experiment

**Fig 4:**
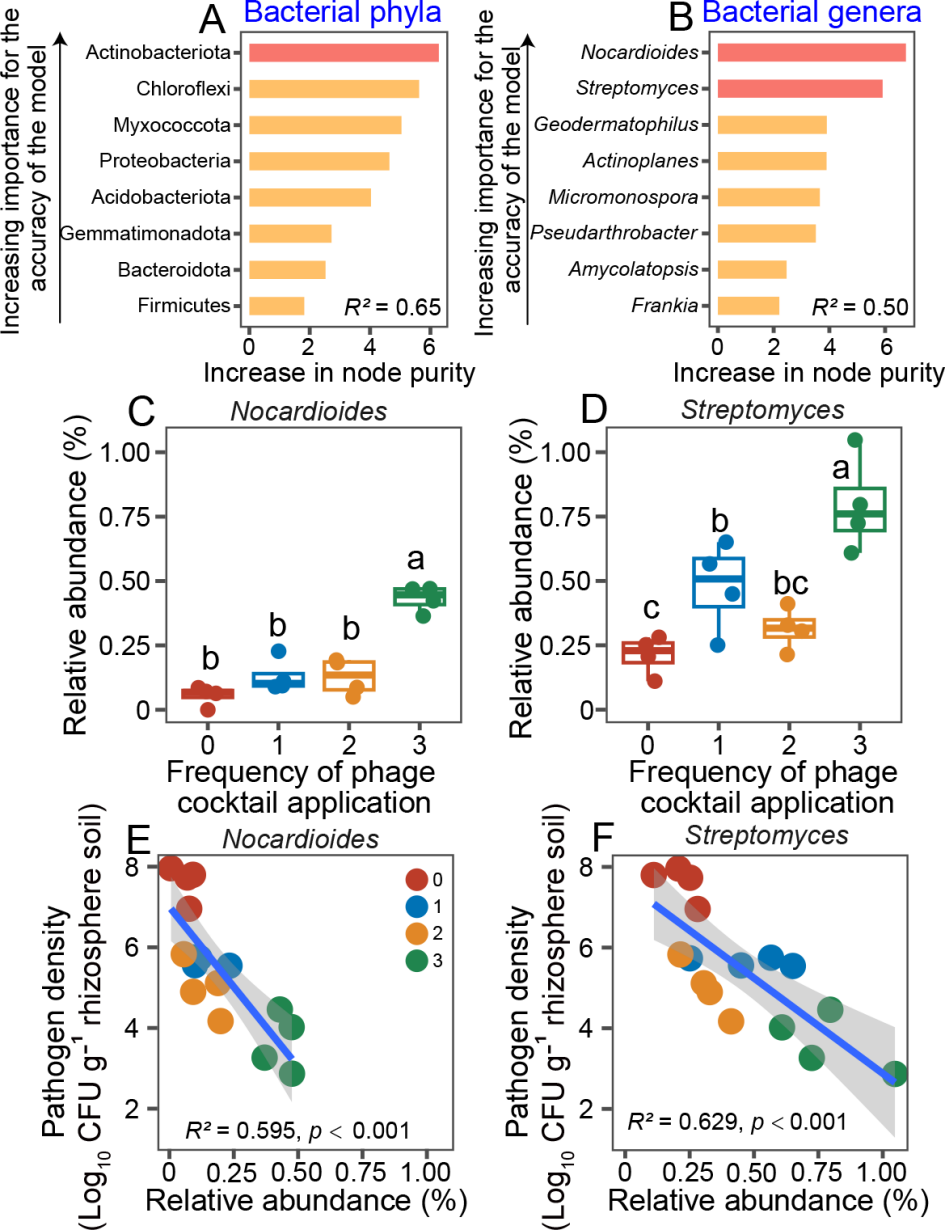
Increase in Actinobacterial taxa abundances is associated with phage-mediated pathogen density reduction in greenhouse experiment. Panels **A** and **B** show the mean predictor importance (increase in node purity, “IncNodePurity”) of eight most abundant phyla and bacterial genera, respectively, based on RF analysis. Predictor taxa that showed higher percentage increases in node purity were considered as relatively more important predictors, and taxa are ranked in descending order of importance based on the accuracy of the model. Panels **C** and **D** show the relative abundances of two most important predictor genera (*Nocardioides* and *Streptomyces*) along with phage application frequency (*N* = 4); lowercase letters and asterisk denote statistically significant differences between treatments (Duncan’s multiple range test). Panels **E** and **F** show significant negative correlations (*P* < 0.05) between pathogen and *Nocardioides* or *Streptomyces* densities (*N* = 4); lines show fitted linear regression and SEM as shaded area around the fitted mean. The red color in panels **C–F** denotes the control treatment, while blue, yellow, and green lines represent the phage applied once, two times, or three times, respectively.

To identify potential pathogen-suppressing bacterial taxa in the resident microbiota in the greenhouse data, we used a random forest analysis and eight most abundant phyla to identify taxa associated with pathogen density reduction. In-line with species indicator analysis, Actinobacteria and Chloroflexi were the most important phyla associated with low pathogen densities (Fig. 4A). As Actinobacteria are known for their antibiotics production and had a higher relative abundance, we conducted RF analysis at the genera level for Actinobacteria phylum (top 7 genera included). The two most important bacterial taxa were *Nocardioides* and *Streptomyces* (Fig. 4B), whose relative abundances increased along with the phage application frequency (*Nocardioides: F*_3,15_ = 33.780, *P* < 0.001; *Streptomyces: F*_3,15_ = 13.523, *P* < 0.001, Fig. 4C and D), and were negatively correlated with pathogen densities (*Nocardioides: R*^2^ = 0.595, *P* < 0.001; *Streptomyces: R*^2^ = 0.629, *P* < 0.001; Fig. 4E and F). The *Nocardioides* and *Streptomyces* were, hence, potential pathogen-suppressing taxa.

### *Nocardioides* or *Streptomyces* are pathogen-suppressing taxa that improve phage cocktail efficacy

**Fig 5:**
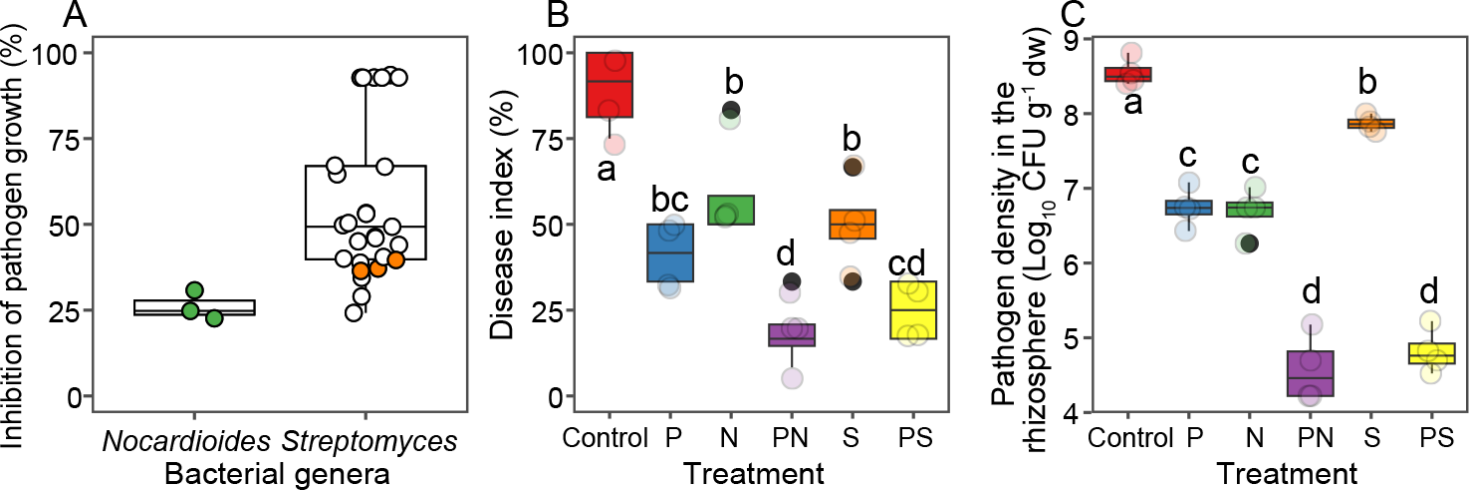
The *Nocardioides* and *Streptomyces* are pathogen-suppressing taxa that improve the phage cocktail efficacy. Panel **A** shows the suppressiveness of 10 actinobacterial isolates for the growth of *R. solanacearum* based on supernatant assay. The green and orange dots represent the *Nocardioides* and *Streptomyces* strains whose suppressiveness was also tested *in vivo* in a separate greenhouse experiment (**B and C**). Panels **B** and **C** show the bacterial wilt disease index and pathogen densities at the end of the greenhouse experiment, respectively. The treatments (*N* = 4) are coded as follows: Control, pathogen only (red); P, phage alone (blue); N, *Nocardioides* alone (green); S, *Streptomyces* alone (purple); PN, phage and *Nocardioides* (orange); and PS, phage and *Streptomyces* (yellow). Lowercase letters denote statistically significant differences between different treatments (Duncan’s multiple range test).

To validate if Actinobacteria were potential pathogen-suppressing taxa, 10 actinobacterial colonies were isolated and purified from the healthy plants of pooled phage treatment samples at the end of the greenhouse experiment. Based on 16S rRNA amplicon sequencing, these 10 colonies consisted of 1 *Nocardioides* and 9 *Streptomyces* strains (Fig. S4). All strains suppressed the growth of *R. solanacearum* in laboratory experiments *in vitro* with varying degrees (*F*_9,29_ = 115.070, *P* < 0.001). Overall, *Streptomyces* strains were more suppressive than *Nocardioides* strain, even though their suppressiveness varied between specific *Streptomyces* strains (*F*_1,29_ = 5.501, *P* = 0.026, Fig. 5A; Fig. S5). The only *Nocardioides* and one randomly selected *Streptomyces* strain were chosen for a separate greenhouse experiment to test their suppressiveness to *R. solanacearum* either individually or in combination with the phage cocktail. When applied individually, phage cocktail, and *Nocardioides* and *Streptomyces* treatments all reduced the disease incidence and pathogen densities in similar degree (disease index: *F*_5,23_ = 17.119, *P* < 0.001; pathogen density: *F*_5,23_ = 119.420, *P* < 0.001, all pairwise comparisons non-significant, Fig. 5B and C). However, both disease incidence and pathogen densities were reduced relatively more when *Nocardioides* or *Streptomyces* strains were combined with the phage cocktail, indicative of synergistic pathogen suppression. Relative to the pathogen-only control treatment, the plant protection efficacy by the phage cocktail improved on average 79% and 72% when combined with *Nocardioides* or *Streptomyces* strain, respectively. Similarly, combining phage cocktail with *Nocardioides* or *Streptomyces* improved pathogen density control by 55% and 40% compared to the phage-only treatment, respectively. Together, these results demonstrate that phage application could potentially trigger a second line of defense by the surrounding bacterial community by increasing the relative abundance of pathogen-suppressing Actinobacteria.

## DISCUSSION

**Fig 6:**
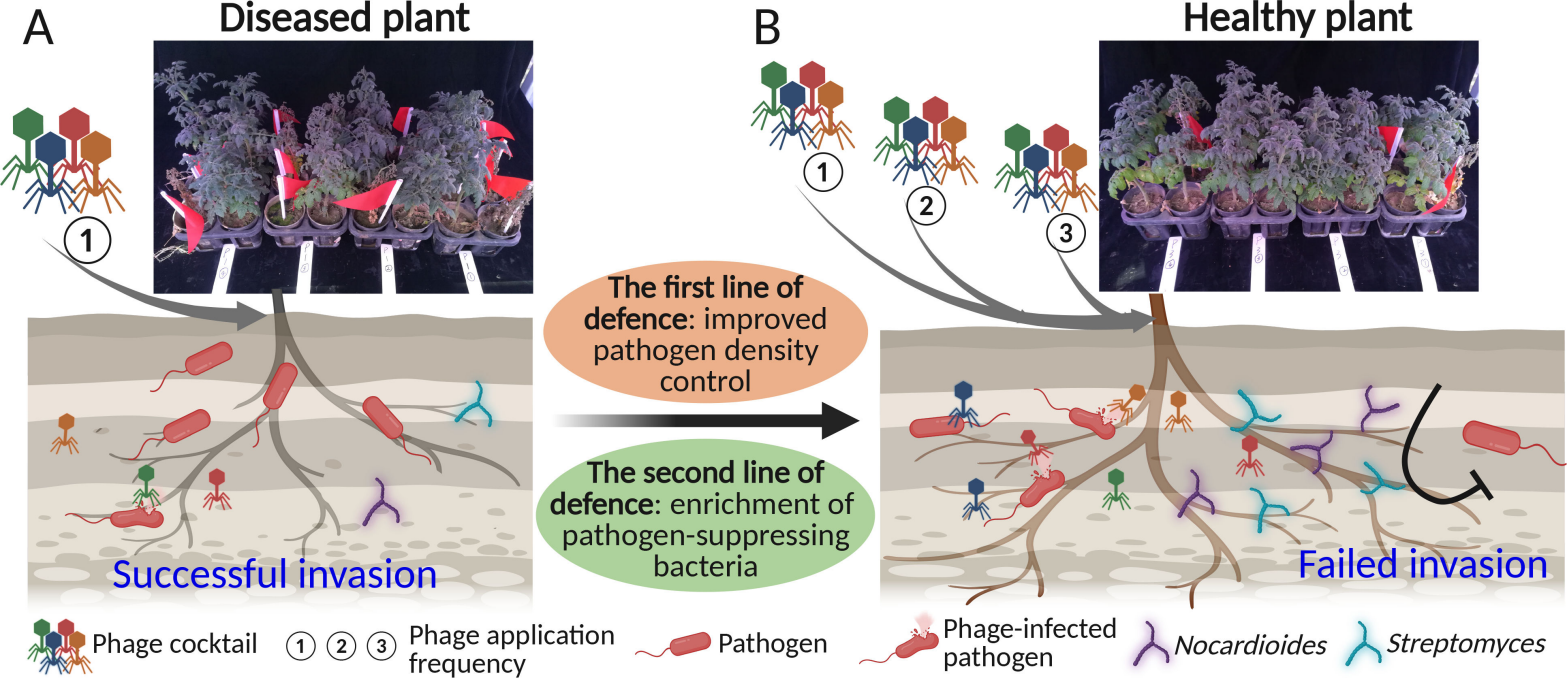
Schematic summarizing how phage cocktail application frequency potentially improves the pathogen biocontrol directly and indirectly. Applying phage cocktail only once (**A**) led to relatively weaker pathogen density control, that is, “the first line of defense,” compared to when phage cocktail was applied for three times (**B**). Applying phage cocktail for three times also led to a clearer increase in the abundance of pathogen-suppressing *Nocardioides* and *Streptomyces* bacterial taxa, activating “the second line of defense” by pre-existing microbiota.

While there is growing interest in using phages for biocontrolling plant bacterial diseases, we still have limited understanding on how phages should be applied to achieve optimal efficacy. To address this, we tested if we can improve phage biocontrol efficacy by applying them repeatedly, and furthermore, if the phage efficacy is dependent on the resident rhizosphere microbiota present in the tomato rhizosphere. We found that repeated phage-cocktail application reduced the pathogen densities and disease incidence most clearly in both greenhouse and field experiments. Crucially, repeated phage application was also associated with clear changes in the resident bacterial microbiota, resulting in enrichment of Actinobacteria that could be used to synergistically improve the efficacy of phage cocktail (Fig. 6). Together, our results show that the phage biocontrol efficacy is microbiome context dependent, which should be acknowledged when developing phage treatments against pathogens that reside in polymicrobial communities.

We found that the efficacy of a phage cocktail could be significantly improved by applying them repeatedly during the tomato growth cycle. In-line with a previous study (12), single phage cocktail application resulted in approximately 33% and 40% reduction in bacterial wilt disease during the greenhouse and field experiments, respectively. In contrast, much improved plant protection was achieved when phages were applied for three times, leading to 67% and 84% reductions in disease incidence in greenhouse and field experiments, respectively. This increase in efficacy can be partially attributed to relatively higher phage densities in the rhizosphere. While the phage densities were reduced by 51% in single phage cocktail application treatments (down to 4 × 10³ PFU g^−1^ soil), phage densities remained constantly high when applied repeatedly for three times (four orders of magnitude higher densities: 2.5 × 10⁷ PFU g^−^¹ soil). The survival and persistence of phages in soils have previously been demonstrated to show notable declines to densities lower than 10⁶ PFU g⁻¹ soil (12, 65, 66). It is thus likely that repeated phage application frequency improved the efficacy of phage biocontrol by increasing phage densities and phage-bacteria encounter rates in the rhizosphere. It is also worth noting that similar to a previous study (12), phage cocktail application was also efficient in the field, even though the level of disease incidence was quite low in this experiment likely due to low environmental temperature during the time of the year (67). Repeated phage cocktail application could, hence, provide a robust way to improve phage biocontrol in temporally and spatially variable conditions typical for agricultural setting.

Previous studies have demonstrated that *R. solanacearum* can quickly evolve resistance to phages both in the lab and in the rhizosphere (12, 44). In contrast to these studies, we did not observe any resistant *R. solanacearum* colonies during the first and second sampling time points (days 8 and 15), and there are several potential explanations for this. First, repeated phage cocktail application reduced the *R. solanacearum* densities below 5 × 10³ CFU g^−1^ in the soil, which did not recover, especially when phages were applied for three times. Low *R. solanacearum* population densities could have constrained phage resistance evolution by reducing the emergence of phage-resistant variants due to low mutation supply rate (68, 69). Second, previous work has demonstrated that phage resistance is costly to *R. solanacearum*, leading to reduced growth and competitiveness (12). Such costs could have constrained the selective advantage of phage-resistant mutants, reducing their frequency in the rhizosphere. Third, using phages as a cocktail could constrain the evolution of resistance if there is no, or only weak, cross-resistance between different phages (23). For example, it has been shown that combining two phages that use either type IV pilus or LPS as their surface receptor constrains the emergence of resistance in *Pseudomonas aeruginosa* because dual-phage, generalist resistance is more rare and more costly (20, 21). While some levels of cross-resistance were previously shown to evolve in *R. solanacearum* when challenged with three-phage cocktail application, the level of such broad range phage resistance was low, likely due to high costs of resistance (12). Even though we cannot conclude that phage resistance would not evolve over longer time scale, our results suggest that continuously high phage selection pressure might constrain the emergence of resistance, thereby promoting the long-term efficacy of phage therapy.

Most importantly, we found that increase in phage biocontrol efficacy was likely dependent on the pre-existing rhizosphere microbiota. First, we found that increase in phage application frequency led to clear changes in the diversity, composition, and relative abundance of resident rhizosphere bacterial taxa. This is in-line with previous research, where phage cocktail application was linked to shifts in the composition and diversity of the resident bacterial microbiota, and enrichment of bacterial taxa that exhibited antagonism or facilitation toward the pathogen (12). Phages could, hence, be potentially used to buffer soil microbiomes from the diversity- and composition-related changes that are often associated with successful pathogen invasions and dominance in the rhizosphere (39, 70). We further discovered that repeated phage cocktail application enriched especially Actinobacteria and Chloroflexi bacteria. The Chloroflexi belong to an oligotrophic phylum renowned for their carbohydrate metabolism (71, 72). Previous research has demonstrated that Chloroflexi can dominate nutrient-deprived environments (73) and hinder the proliferation of copiotrophic microbes through resource competition (74). It is, hence, possible that phage-mediated reduction in *R. solanacearum* abundances helped Chloroflexi to take up vacant resource niche space, leading to increase in their relative abundance. Another group that responded positively to phage application frequency was Actinobacteria that are renowned for their diverse secondary metabolism and production of antimicrobial compounds that can suppress soil-borne pathogens (75, 76). The benefits of Actinobacteria on plant health have been extensively investigated (77), and their abundances have been linked to enhanced immune activation in plants and direct inhibition of *R. solanacearum* (78, 79). We found that especially two Actinobacteria genera, *Streptomyces* and *Nocardioides*, were enriched in the rhizosphere when phages were applied more frequently. All isolates of these genera showed clear *R. solanacearum* suppression *in vitro* and *in vivo*, while this effect varied considerably between different *Streptomyces* isolates, which could be attributed to their inherent metabolic versatility (75, 80, 81). For example, *S. panaciradicis* produces actinomycin D, which has antibacterial activity against *R. solanacearum* (82), while *Streptomyces* strain UT4A49 and *S. koyangensis* strain VK-A60 produce 2,4-di-tert-butylphenol and 4-phenyl-3-butenoic acid, respectively, which also inhibit the growth of *R. solanacearum* (83, 84). Differences in the antimicrobial activity and produced quantities of these compounds could potentially explain observed differences in the suppressiveness of tested *Streptomyces* strains (79). While *Nocardioides* are primarily known for their ability to degrade organic compounds, some strains have recently shown to have antifungal and plant growth-promoting activity (85, 86). For example, *N. thermolilacinus* strain SON-17 can reduce disease severity by inhibiting the germination of spores of pathogenic fungi (87), while actinomycin produced by *N. luteus* has shown promising activity against some pathogenic fungi and bacteria (88). Even though the single *Nocardioides* sp. isolate showed a poor inhibition via antibiosis, it performed well in a greenhouse experiment, indicative of non-antimicrobial suppression of *R. solanacearum*. For example, the production of siderophores, competition for nutrients, or induction of systemic plant resistance has not yet been reported as mechanisms of biocontrol by non-*Streptomyces* Actinomycetes (89) and should be investigated in more detail in the future. Despite the potential difference in the mode of *R. solanacearum* suppression, both *Nocardioides* and *Streptomyces* strains were effective in pathogen and bacterial wilt disease control, especially when combined with phages, indicative of synergistic pathogen suppression. This is in-line with our previous study where we observed a similar synergistic effect between *Bacillus amyloliquefaciens* and phage by increasing pathogen susceptibility to antibiotics (44). The phenomenon, known as phage-antibiotic synergy (PAS), has been documented in the treatment of bacterial infections in both agricultural and clinical contexts (90–92). In this experiment, *Bacillus* species were not clearly associated with the pathogen density reduction, which suggests that perhaps the identity of pathogen-suppressing bacteria might not be that important for the suppression. Instead, potential PAS effects could be achieved with several different taxa as long as they are able to compete with the pathogen either through shared resources or via production of antimicrobial compounds. In the future, it would be interesting to study if even better pathogen suppression can be attained by repeatedly applying both phages and pathogen-suppressing taxa in combination. Together, these findings highlight the potential for elevating the biocontrol effectiveness of phage cocktails through two avenues: integrating suppressive bacterial species within the cocktail itself or enhancing the activity of these antagonistic bacterial species already present within the agricultural soils.

To conclude, our study shows that repeated phage cocktail application can improve the biocontrol efficacy of bacterial wilt disease and trigger soil suppressiveness via recruitment of pathogen-suppressive microbial taxa. Future investigations are needed to elucidate the potential molecular mechanisms underlying the synergy between phages and pathogen-suppressive bacterial taxa and how they might shape microbiome dynamics beyond the pathogen and Actinobacteria. As phage-antibiotic synergies are often observed with clinical pathogens, it is likely that our findings hold also in animal and human microbiomes, where phage therapies could be designed to take advantage of beneficial host resident microbiota. For example, other studies have also shown that phage application can change the microbial community composition in aquatic microbiomes (93) and the mouse gut microbiota (94). Finally, further technological development is required to translate phage application techniques across hydroponic, aeroponic, and drip irrigation agricultural systems to optimize phage inoculation techniques and timing in greenhouse and field conditions.

## Data Availability

The data sets generated during and/or analyzed during the current study are available in the dryad digital repository, doi: 10.5061/dryad.dz08kps40. The raw reads of 16S rRNA amplicon sequencing were deposited into the NCBI Sequence Read Archive (SRA) database (Accession Number: PRJNA819579).

## SUPPLEMENTAL MATERIAL

Supplemental figures and tables. mbio.03016-23-s0001.docx Fig. S1 to S5; Table S1 to S3.

ASM does not own the copyrights to Supplemental Material that may be linked to, or accessed through, an article. The authors have granted ASM a non-exclusive, world-wide license to publish the Supplemental Material files. Please contact the corresponding author directly for reuse.
