# Supplementary material for: Phages enhance both phytopathogen density control and rhizosphere microbiome suppressiveness
Source: mBio. 2024 May 23;15(6):e03016-23. doi: 10.1128/mbio.03016-23 (PMC11237578; doi:10.1128/mbio.03016-23)
Supplement: Supplemental figures and tables. — Fig. S1 to S5; Table S1 to S3. [file mbio.03016-23-s0001.docx]

Title: Phages enhance both phytopathogen density control and rhizosphere microbiome suppressiveness

Xiaofang Wang^1,#^, Shuo Wang^1,#^, Keming Yang^1,2^, Mingcong Huang^1^, Yilin He^1^, Ningqi Wang^1^, Tianyu Sun^1^, Hongwu Yang^3^, Tianjie Yang^1^, Yangchun Xu^1^, Qirong Shen^1^, Ville-Petri Friman^1,4,^*, Zhong Wei^1,^*

^1^Key Lab of Organic-based Fertilizers of China and Jiangsu Provincial Key Lab for Solid Organic Waste Utilization, Nanjing Agricultural University, Nanjing 210095, China

^2^ College of Agro-grassland Science, Nanjing Agricultural University, Nanjing 210095, Jiangsu, P.R. China

^3^China National Tobacco Corporation Hunan Company, Changsha 410005, Hunan, P.R. China

^4^Department of Microbiology, University of Helsinki, 00014, Helsinki, Finland

^#^ These authors contribute equally to this work

*Correspondence: weizhong@njau.edu.cn (Zhong Wei); ville-petri.friman@helsinki.fi (Ville-Petri Friman)

**Supplemental Figures**


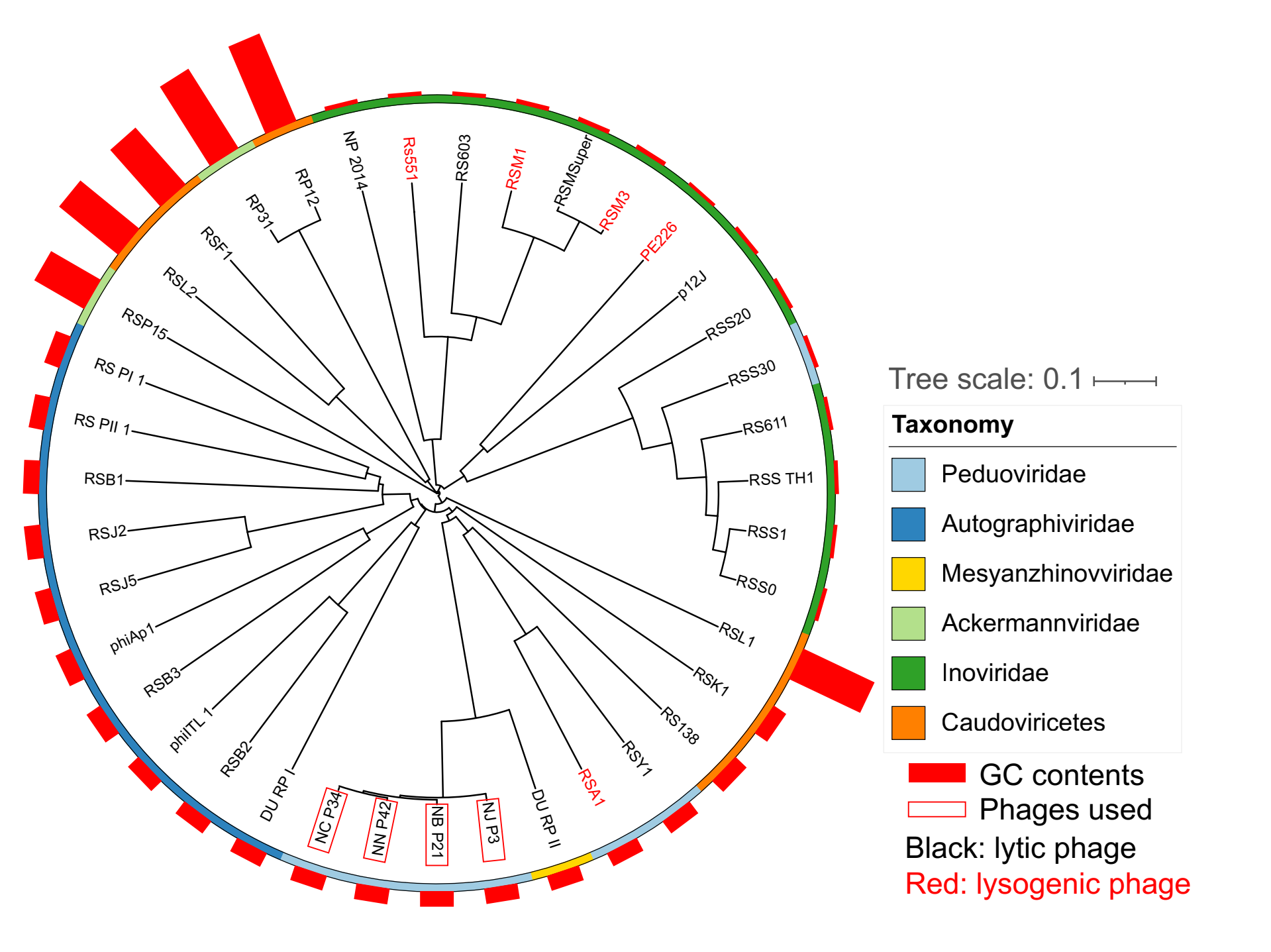


**Figure S1.** Phylogenetic tree showing the relatedness of lytic and lysogenic phages based on genetic similarity. Each genome was first compared to another and to themselves to account and normalize for differences in phage genomes sizes and a neighbour joining tree was then constructed based on the complete distance matrix based on 999 bootstrap sample values as described previously (1). Known lytic and lysogenic phages are shown in black and red, respectively. The phages used in this experiment (NJ-P3, NB-P21, NC-P34, NN-P42; bottom of the figure with red rectangular outline) belong to *Peduviridae* family and were most closely related to lytic phage DU_RP_II.


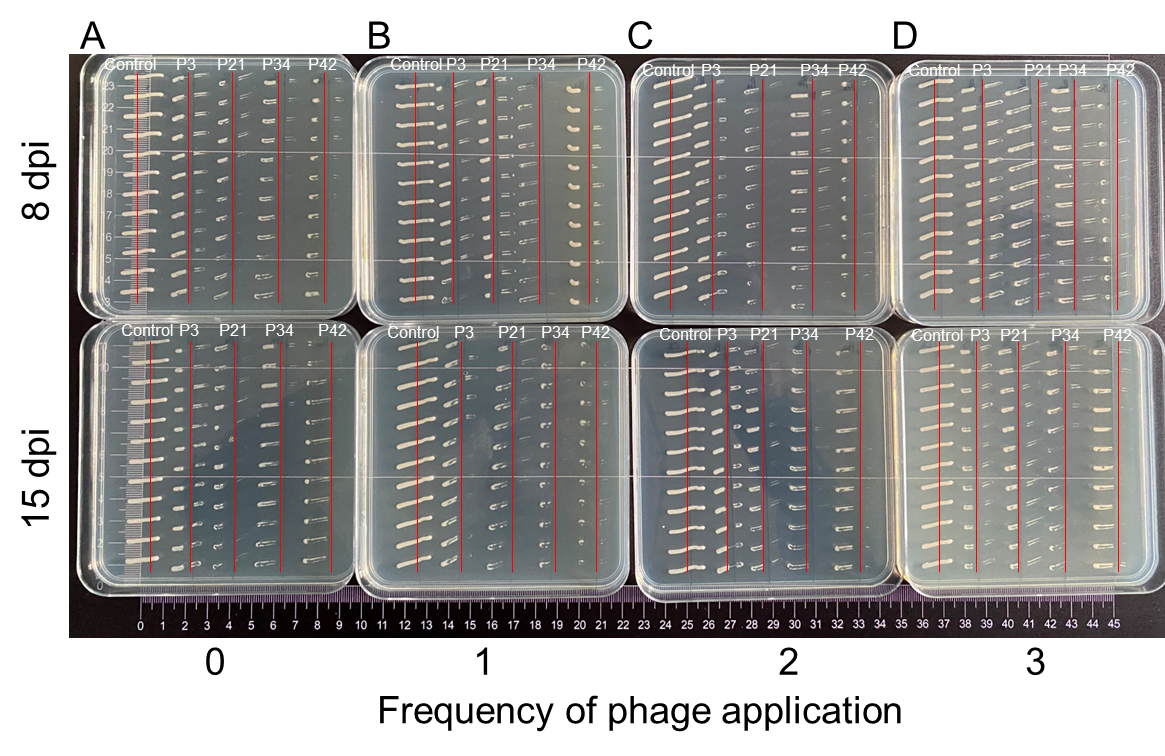


**Figure S2.** Phage resistance assay quantifying *R. solanacearum* pathogen sensitivity to each of the four ancestral phages on streak plate assay. Three *R. solanacearum* colonies from each treatment replicate soil samples were isolated after 8 and 15 days from pathogen inoculation, resulting 12 colonies per treatment and timepoint. Inhibition of *R. solanacearum* growth over the phage line (on red) is indicative of susceptibility to a given ancestral phage. All isolated bacterial were susceptible to all four ancestral phages. Water was used as negative control (Control; leftmost column on each square plate) and tested phages (from left to right in columns 2-5 on each square plate) were: NJ-P3, NB-P21, NC-P34 and NN-P42. Panels A-D denote for phage application frequency treatments: A: no phage added, B: phage applied once, C: phage applied for two times and D: phage applied for three times; top and bottom rows show colonies isolated after 8 and 15 days post inoculation (dpi).


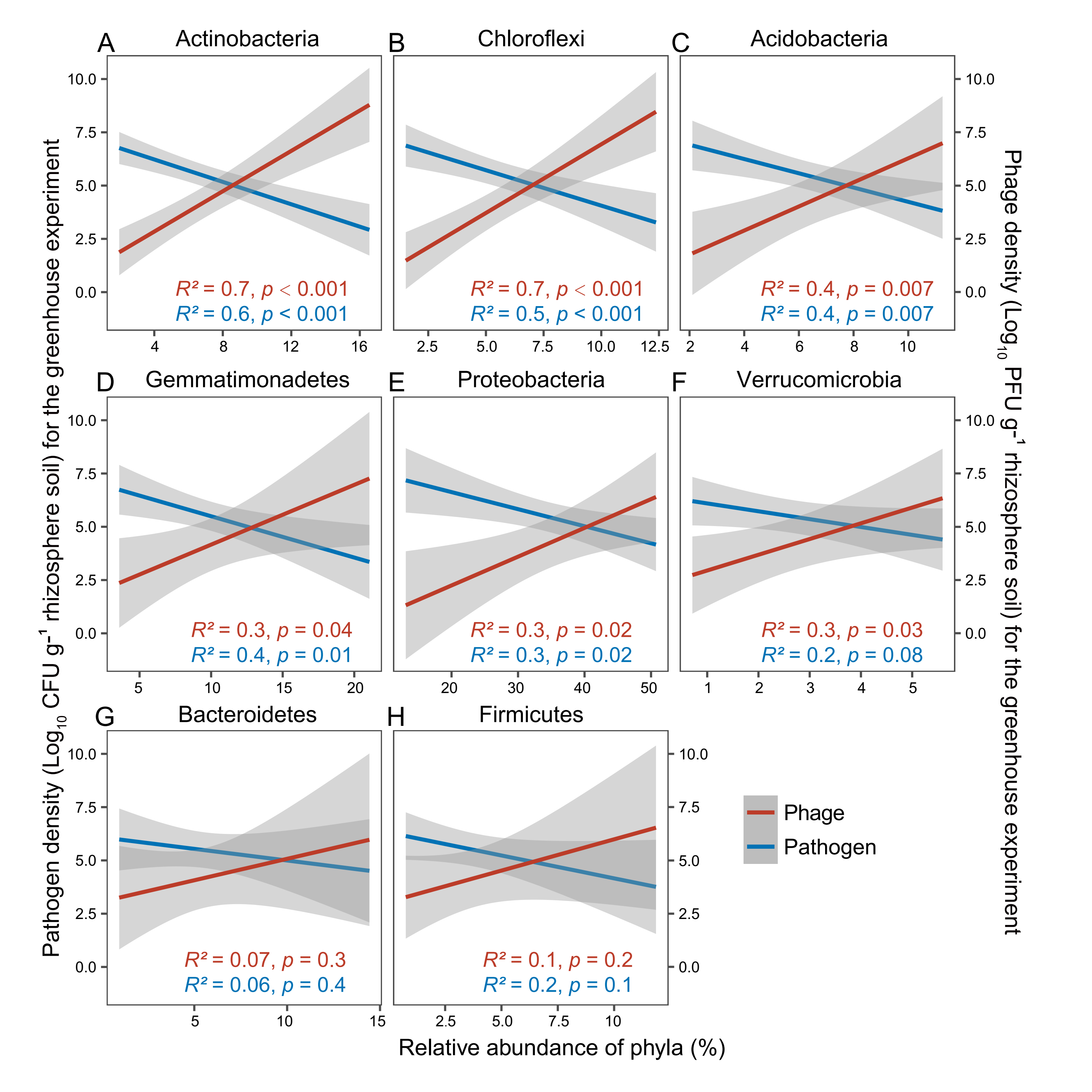


**Figure S3.** Certain bacterial phyla abundances correlated positively with pathogen and phage densities in the rhizosphere. Lines in all panels show fitting of linear regression. The data is averaged across all phage treatments.


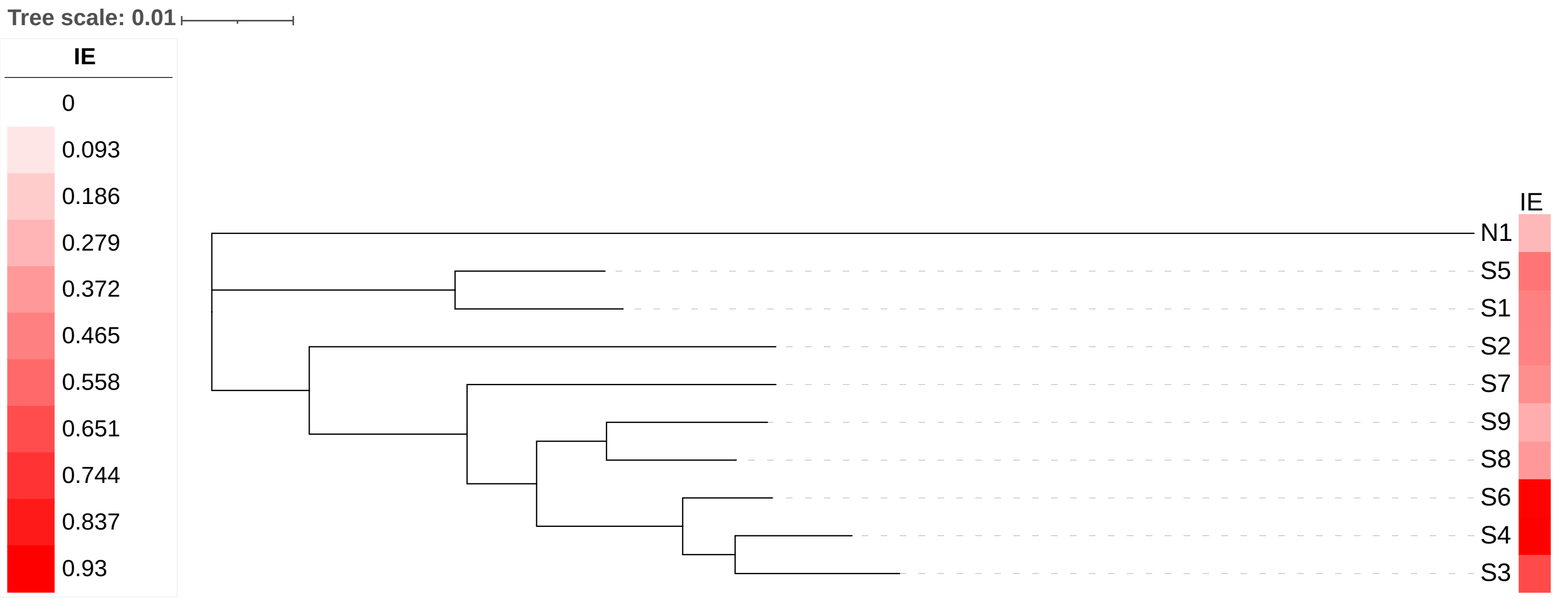


**Figure S4.** Neighbor-joining phylogenetic tree of 10 *Actinomyces* strains based on 16S rRNA gene sequence similarity. Heatmap on the right side of the tree shows the effect of these 10 strains on *R. solanacearum* pathogen growth inhibition measured using supernatant assay in the lab (N = 3).


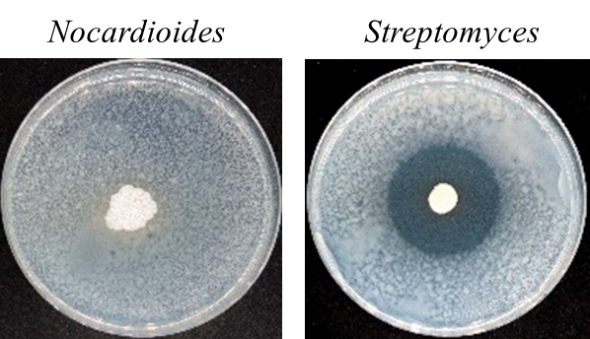


**Figure S5.** The inhibition halos of *R. solanacearum* on agar plates that were spotted with *Nocardioides* (left) or *Streptomyces* (right) strains in the middle (two strains used in the greenhouse validation experiment).

**Supplemental tables**

**Table S1**. Table showing the name, location, year of isolation and morphology of bacteriophages used in this study.

| Name | Location and year of isolation | Taxonomic classification |
| --- | --- | --- |
| NJ-P3 | Tomato rhizosphere, Nanjing, China (118°57' E, 32°03' N), 2015 | Peduviridae family |
| NB-P21 | Tomato rhizosphere, Ningbo, China (121°67' E, 29°91' N), 2015 | Peduviridae family |
| NC-P34 | Tomato rhizosphere, Nanchang, China (115°51′E, 28°41′N), 2015 | Peduviridae family |
| NN-P42 | Tomato rhizosphere, Nanning, China (108°21′E, 22°49′N), 2015 | Peduviridae family |

**Table S2**. Indicator species identified in different phage cocktail application frequency treatments (1=identified as indicator species; 0=not identified as indicator species)

| **nodes_id** | **Frequency of phage cocktail application** | | | | **stat** | **p-value** |
| --- | --- | --- | --- | --- | --- | --- |
|  | **0** | **1** | **2** | **3** |  |  |
| Genus30 | 0 | 0 | 1 | 1 | 0.7676 | 0.0070 |
| Genus43 | 0 | 0 | 0 | 1 | 0.8753 | 0.0010 |
| Genus53 | 0 | 0 | 1 | 1 | 0.7784 | 0.0090 |
| Genus77 | 0 | 0 | 0 | 1 | 0.7750 | 0.0010 |
| Genus89 | 0 | 0 | 0 | 1 | 0.9247 | 0.0010 |
| Genus97 | 0 | 0 | 0 | 1 | 0.7922 | 0.0040 |
| Genus106 | 0 | 0 | 0 | 1 | 0.8664 | 0.0010 |
| Genus107 | 0 | 0 | 0 | 1 | 0.8293 | 0.0010 |
| Genus109 | 0 | 0 | 0 | 1 | 0.8271 | 0.0010 |
| Genus112 | 0 | 0 | 0 | 1 | 0.7839 | 0.0030 |
| Genus141 | 0 | 1 | 0 | 0 | 0.9094 | 0.0050 |
| Genus171 | 0 | 0 | 0 | 1 | 0.7651 | 0.0050 |
| Genus176 | 0 | 1 | 1 | 0 | 0.8449 | 0.0030 |
| Genus222 | 0 | 0 | 0 | 1 | 0.8580 | 0.0010 |
| Genus233 | 0 | 0 | 0 | 1 | 0.8980 | 0.0010 |
| Genus248 | 0 | 0 | 0 | 1 | 0.7627 | 0.0050 |
| Genus262 | 0 | 0 | 1 | 0 | 0.7463 | 0.0030 |
| Genus264 | 0 | 0 | 1 | 0 | 0.7694 | 0.0080 |
| Genus276 | 0 | 0 | 0 | 1 | 0.8631 | 0.0040 |
| Genus289 | 0 | 0 | 0 | 1 | 0.8855 | 0.0010 |
| Genus299 | 0 | 0 | 0 | 1 | 0.7637 | 0.0070 |
| Genus343 | 0 | 1 | 1 | 1 | 0.7253 | 0.0100 |
| Genus353 | 0 | 0 | 1 | 0 | 0.8221 | 0.0070 |
| Genus371 | 0 | 0 | 1 | 1 | 0.7344 | 0.0080 |
| Genus377 | 0 | 1 | 1 | 1 | 0.8854 | 0.0040 |
| Genus381 | 0 | 1 | 1 | 1 | 0.7985 | 0.0040 |
| Genus438 | 0 | 0 | 0 | 1 | 0.8504 | 0.0010 |
| Genus441 | 0 | 0 | 1 | 0 | 0.8299 | 0.0080 |
| Genus450 | 0 | 1 | 0 | 0 | 0.8062 | 0.0080 |
| Genus458 | 0 | 1 | 1 | 1 | 0.7118 | 0.0070 |
| Genus459 | 0 | 0 | 0 | 1 | 0.7864 | 0.0060 |
| Genus493 | 0 | 0 | 0 | 1 | 0.7174 | 0.0100 |
| Genus503 | 0 | 0 | 1 | 1 | 0.7521 | 0.0070 |
| Genus522 | 0 | 0 | 1 | 1 | 0.8748 | 0.0010 |
| Genus526 | 0 | 1 | 1 | 1 | 0.8131 | 0.0040 |
| Genus543 | 1 | 0 | 0 | 0 | 0.8089 | 0.0020 |
| Genus563 | 1 | 0 | 0 | 0 | 0.9680 | 0.0040 |
| Genus566 | 0 | 1 | 0 | 0 | 0.8512 | 0.0050 |
| Genus596 | 0 | 1 | 1 | 1 | 0.7579 | 0.0090 |
| Genus600 | 0 | 1 | 1 | 1 | 0.8591 | 0.0040 |
| Genus607 | 0 | 1 | 0 | 0 | 0.7765 | 0.0060 |
| Genus624 | 0 | 1 | 1 | 1 | 0.7701 | 0.0060 |
| Genus629 | 0 | 0 | 1 | 0 | 0.6982 | 0.0020 |
| Genus643 | 0 | 0 | 0 | 1 | 0.7500 | 0.0060 |
| Genus671 | 0 | 0 | 0 | 1 | 0.7073 | 0.0080 |
| Genus716 | 0 | 0 | 1 | 1 | 0.8431 | 0.0010 |
| Genus724 | 0 | 0 | 1 | 0 | 0.7368 | 0.0020 |

**Table S3.** Bacterial co-occurrence network data across all treatment samples

| **nodes_id** | **node_degree** | **node_betw** | **node_evcent** | **Clustering_coefficient** | **No.module** | **Zi** | **Pi** |
| --- | --- | --- | --- | --- | --- | --- | --- |
| Genus458 | 7 | 302.8837 | 0.0021 | 0.5238 | 1 | 2.44458 | 0.5266 |
| Genus600 | 12 | 912.1580 | 0.0076 | 0.3030 | 1 | -0.04242 | 0.2781 |
| Genus624 | 11 | 965.2206 | 0.0083 | 0.3273 | 1 | 0.58908 | 0.2602 |
| Genus596 | 3 | 78.4933 | 0.0015 | 0.3333 | 1 | -0.01310 | 0.3600 |
| Genus8 | 12 | 396.2484 | 0.0019 | 0.4091 | 1 | 2.24132 | 0.0635 |
| Genus26 | 9 | 55.1708 | 0.0018 | 0.5833 | 1 | -0.04242 | 0.5375 |
| Genus11 | 12 | 568.0287 | 0.0102 | 0.3182 | 1 | 0.00000 | 0.0000 |
| Genus115 | 9 | 718.7020 | 0.0154 | 0.3333 | 1 | -1.15965 | 0.0000 |
| Genus49 | 11 | 410.4275 | 0.0033 | 0.3273 | 1 | -1.26775 | 0.0000 |
| Genus209 | 13 | 348.7157 | 0.0031 | 0.3205 | 1 | -1.00400 | 0.6000 |
| Genus169 | 3 | 335.2833 | 0.0001 | 0.0000 | 1 | 2.00093 | 0.0678 |
| Genus230 | 5 | 608.2555 | 0.0006 | 0.0000 | 1 | -0.88894 | 0.6875 |
| Genus281 | 7 | 248.0133 | 0.0014 | 0.5238 | 1 | 0.27505 | 0.4694 |
| Genus345 | 11 | 894.4161 | 0.0011 | 0.2545 | 1 | 0.43837 | 0.6433 |
| Genus347 | 13 | 858.8933 | 0.0040 | 0.3590 | 1 | -0.76361 | 0.5469 |
| Genus352 | 8 | 231.0487 | 0.0022 | 0.4286 | 1 | 0.00000 | 0.7500 |
| Genus356 | 11 | 354.9288 | 0.0033 | 0.4182 | 1 | -0.95027 | 0.6875 |
| Genus379 | 7 | 53.8932 | 0.0069 | 0.5714 | 1 | 2.00390 | 0.2781 |
| Genus395 | 1 | 0.0000 | 0.0003 | NA | 1 | -0.41272 | 0.6800 |
| Genus413 | 11 | 608.9405 | 0.0063 | 0.3273 | 1 | 1.85981 | 0.4219 |
| Genus433 | 5 | 128.1854 | 0.0012 | 0.5000 | 1 | 0.00000 | 0.0000 |
| Genus367 | 11 | 1036.4316 | 0.0095 | 0.2909 | 1 | -0.58938 | 0.5556 |
| Genus118 | 2 | 18.0112 | 0.0000 | 0.0000 | 1 | -0.02986 | 0.5621 |
| Genus464 | 2 | 0.0000 | 0.0001 | 1.0000 | 1 | 1.35657 | 0.6500 |
| Genus384 | 6 | 817.5464 | 0.0006 | 0.2000 | 1 | -0.61237 | 0.0000 |
| Genus119 | 1 | 0.0000 | 0.0004 | NA | 1 | -0.57735 | 0.0000 |
| Genus121 | 1 | 0.0000 | 0.0000 | NA | 1 | -0.87753 | 0.7778 |
| Genus612 | 2 | 0.0000 | 0.0000 | 1.0000 | 1 | -1.16567 | 0.5556 |
| Genus93 | 1 | 0.0000 | 0.0001 | NA | 1 | -0.15316 | 0.5556 |
| Genus548 | 11 | 289.4940 | 0.0015 | 0.4000 | 1 | -0.16262 | 0.5111 |
| Genus689 | 2 | 246.1015 | 0.0012 | 0.0000 | 1 | 0.19797 | 0.1378 |
| Genus358 | 3 | 38.7567 | 0.0020 | 0.3333 | 1 | 0.55856 | 0.4014 |
| Genus406 | 3 | 0.0000 | 0.0005 | 1.0000 | 1 | -1.15965 | 0.5556 |
| Genus490 | 3 | 209.6720 | 0.0009 | 0.3333 | 1 | -0.30124 | 0.4375 |
| Genus492 | 3 | 59.9562 | 0.0065 | 0.0000 | 1 | 1.13947 | 0.3500 |
| Genus589 | 2 | 159.8504 | 0.0003 | 0.0000 | 1 | -1.16567 | 0.7500 |
| Genus599 | 4 | 4.3914 | 0.0012 | 0.8333 | 1 | 0.58908 | 0.5930 |
| Genus622 | 4 | 270.3392 | 0.0000 | 0.1667 | 1 | 1.20802 | 0.1142 |
| Genus675 | 4 | 0.0000 | 0.0016 | 1.0000 | 1 | -0.49407 | 0.3056 |
| Genus678 | 10 | 446.9868 | 0.0025 | 0.4222 | 1 | -0.02986 | 0.3500 |
| Genus680 | 2 | 0.0000 | 0.0001 | 1.0000 | 1 | 2.29117 | 0.3048 |
| Genus691 | 1 | 0.0000 | 0.0000 | NA | 1 | -0.88380 | 0.3600 |
| Genus693 | 5 | 30.3456 | 0.0012 | 0.6000 | 1 | 1.71576 | 0.2986 |
| Genus699 | 3 | 45.8734 | 0.0059 | 0.3333 | 1 | 1.15955 | 0.2309 |
| Genus629 | 4 | 0.7578 | 0.0093 | 0.8333 | 2 | 1.83712 | 0.3600 |
| Genus5 | 3 | 0.0000 | 0.0003 | 1.0000 | 2 | 0.19797 | 0.2444 |
| Genus3 | 20 | 389.0251 | 0.0671 | 0.4526 | 2 | -1.36459 | 0.0000 |
| Genus6 | 8 | 598.3923 | 0.0043 | 0.3214 | 2 | 0.91915 | 0.2459 |
| Genus9 | 19 | 288.0427 | 0.1140 | 0.5263 | 2 | 0.31817 | 0.1289 |
| Genus12 | 11 | 304.4065 | 0.0225 | 0.5818 | 2 | 0.85133 | 0.5903 |
| Genus31 | 23 | 756.2850 | 0.0840 | 0.3953 | 2 | -0.87753 | 0.7500 |
| Genus94 | 20 | 637.9495 | 0.1049 | 0.4211 | 2 | -0.95027 | 0.0000 |
| Genus70 | 22 | 439.9582 | 0.1262 | 0.4113 | 2 | -1.36459 | 0.0000 |
| Genus134 | 19 | 513.8261 | 0.1026 | 0.4503 | 2 | 0.06350 | 0.4375 |
| Genus113 | 9 | 58.5533 | 0.0286 | 0.5556 | 2 | 0.00000 | 0.0000 |
| Genus173 | 21 | 326.8231 | 0.1261 | 0.4190 | 2 | -0.95827 | 0.6800 |
| Genus66 | 2 | 7.5563 | 0.0042 | 0.0000 | 2 | -0.40301 | 0.5571 |
| Genus175 | 30 | 1098.2531 | 0.1172 | 0.3770 | 2 | -0.76361 | 0.4694 |
| Genus208 | 17 | 717.6875 | 0.0766 | 0.3750 | 2 | -0.52321 | 0.4700 |
| Genus217 | 12 | 150.3189 | 0.0336 | 0.3636 | 2 | 0.55856 | 0.2068 |
| Genus158 | 6 | 1317.3948 | 0.0041 | 0.1333 | 2 | 1.03935 | 0.1715 |
| Genus224 | 14 | 126.2076 | 0.0464 | 0.5604 | 2 | 0.31817 | 0.5552 |
| Genus219 | 9 | 243.4444 | 0.0347 | 0.4444 | 2 | -0.28282 | 0.1900 |
| Genus273 | 14 | 520.5070 | 0.0425 | 0.3407 | 2 | -1.24439 | 0.7500 |
| Genus268 | 18 | 1132.5073 | 0.0613 | 0.3987 | 2 | -0.28282 | 0.3223 |
| Genus184 | 5 | 294.3558 | 0.0040 | 0.3000 | 2 | 0.56319 | 0.4219 |
| Genus417 | 2 | 2.2505 | 0.0050 | 0.0000 | 2 | -1.15965 | 0.0000 |
| Genus202 | 1 | 0.0000 | 0.0018 | NA | 2 | 1.42762 | 0.3223 |
| Genus422 | 4 | 4.9950 | 0.0117 | 0.6667 | 2 | -0.87753 | 0.7778 |
| Genus33 | 2 | 0.2332 | 0.0041 | 0.0000 | 2 | -0.76361 | 0.3056 |
| Genus223 | 8 | 201.1129 | 0.0152 | 0.5714 | 2 | -0.61237 | 0.0000 |
| Genus227 | 18 | 324.3501 | 0.0790 | 0.5163 | 2 | -0.95027 | 0.7778 |
| Genus423 | 8 | 11.7051 | 0.0364 | 0.7143 | 2 | -0.61237 | 0.0000 |
| Genus428 | 13 | 818.1589 | 0.0368 | 0.2308 | 2 | -0.95827 | 0.7600 |
| Genus214 | 1 | 0.0000 | 0.0020 | NA | 2 | 0.56319 | 0.5702 |
| Genus427 | 9 | 396.6029 | 0.0206 | 0.2778 | 2 | -0.12670 | 0.7500 |
| Genus468 | 31 | 718.5079 | 0.1711 | 0.3978 | 2 | -0.61237 | 0.6875 |
| Genus469 | 12 | 731.0362 | 0.0531 | 0.6515 | 2 | -0.30124 | 0.6000 |
| Genus419 | 4 | 233.0000 | 0.0003 | 0.5000 | 2 | 1.35657 | 0.3056 |
| Genus432 | 10 | 275.0553 | 0.0412 | 0.4222 | 2 | -0.61237 | 0.0000 |
| Genus510 | 14 | 287.4682 | 0.0620 | 0.5275 | 2 | -0.88894 | 0.8125 |
| Genus529 | 23 | 266.9632 | 0.1139 | 0.4822 | 2 | 0.27961 | 0.2986 |
| Genus573 | 12 | 754.8809 | 0.0331 | 0.3939 | 2 | 1.13947 | 0.4545 |
| Genus608 | 3 | 0.0000 | 0.0060 | 1.0000 | 2 | 0.31817 | 0.5990 |
| Genus366 | 5 | 250.5077 | 0.0184 | 0.5000 | 2 | 1.51749 | 0.3686 |
| Genus531 | 4 | 510.1171 | 0.0050 | 0.3333 | 2 | 1.27975 | 0.5023 |
| Genus667 | 5 | 694.0310 | 0.0144 | 0.5000 | 2 | 1.71576 | 0.1736 |
| Genus683 | 12 | 755.4597 | 0.0391 | 0.4091 | 2 | -0.65640 | 0.7500 |
| Genus715 | 7 | 438.2562 | 0.0129 | 0.3810 | 2 | -1.16567 | 0.7500 |
| Genus429 | 18 | 477.0557 | 0.0681 | 0.4314 | 2 | -0.65640 | 0.7500 |
| Genus706 | 17 | 138.5785 | 0.0707 | 0.5735 | 2 | -0.87753 | 0.7778 |
| Genus431 | 8 | 133.1710 | 0.0157 | 0.3929 | 2 | -0.30124 | 0.4375 |
| Genus722 | 10 | 656.5953 | 0.0240 | 0.5556 | 2 | -0.18460 | 0.2344 |
| Genus723 | 28 | 653.5178 | 0.1241 | 0.3968 | 2 | 1.13947 | 0.5208 |
| Genus514 | 5 | 5.3869 | 0.0245 | 0.5000 | 2 | -0.87753 | 0.7500 |
| Genus521 | 1 | 0.0000 | 0.0000 | NA | 2 | 0.27961 | 0.2986 |
| Genus537 | 1 | 0.0000 | 0.0030 | NA | 2 | -0.88894 | 0.8125 |
| Genus639 | 4 | 39.7816 | 0.0098 | 0.1667 | 2 | 0.67876 | 0.1967 |
| Genus717 | 5 | 116.9918 | 0.0021 | 0.6000 | 2 | 0.67876 | 0.1080 |
| Genus719 | 6 | 295.4381 | 0.0081 | 0.3333 | 2 | 0.12487 | 0.6620 |
| Genus730 | 21 | 275.4051 | 0.0706 | 0.4571 | 2 | 0.58908 | 0.5775 |
| Genus264 | 14 | 193.0087 | 0.1032 | 0.5604 | 3 | -1.11301 | 0.7500 |
| Genus43 | 25 | 545.7739 | 0.1620 | 0.4733 | 3 | -0.01310 | 0.6790 |
| Genus89 | 18 | 722.1890 | 0.0655 | 0.4706 | 3 | -0.12670 | 0.7500 |
| Genus77 | 5 | 34.6674 | 0.0298 | 0.6000 | 3 | 1.52043 | 0.4375 |
| Genus97 | 11 | 45.3336 | 0.0279 | 0.6909 | 3 | 0.69686 | 0.5556 |
| Genus106 | 22 | 350.9111 | 0.1490 | 0.5195 | 3 | -0.12670 | 0.7778 |
| Genus107 | 31 | 1345.1828 | 0.1709 | 0.3978 | 3 | -0.12670 | 0.7500 |
| Genus109 | 15 | 504.2799 | 0.0522 | 0.4571 | 3 | 1.35657 | 0.3056 |
| Genus222 | 5 | 231.7545 | 0.0087 | 0.2000 | 3 | 0.74382 | 0.1378 |
| Genus233 | 22 | 922.8576 | 0.0921 | 0.3247 | 3 | 1.01593 | 0.7398 |
| Genus276 | 12 | 45.0927 | 0.0815 | 0.6818 | 3 | 1.13947 | 0.2099 |
| Genus289 | 10 | 159.3441 | 0.0475 | 0.4889 | 3 | -0.16262 | 0.4643 |
| Genus438 | 8 | 18.9504 | 0.0199 | 0.6071 | 3 | -1.36459 | 0.0000 |
| Genus459 | 24 | 1091.0841 | 0.0905 | 0.4058 | 3 | 1.52043 | 0.4375 |
| Genus493 | 13 | 390.8463 | 0.0307 | 0.4359 | 3 | 0.00000 | 0.0000 |
| Genus671 | 33 | 992.8397 | 0.2039 | 0.3655 | 3 | 1.15955 | 0.2864 |
| Genus171 | 1 | 0.0000 | 0.0026 | NA | 3 | -0.87753 | 0.7500 |
| Genus299 | 2 | 1.6592 | 0.0060 | 0.0000 | 3 | -0.80354 | 0.4375 |
| Genus381 | 5 | 605.4430 | 0.0051 | 0.3000 | 3 | -0.52321 | 0.6582 |
| Genus53 | 33 | 717.8002 | 0.2269 | 0.4129 | 3 | -1.16567 | 0.0000 |
| Genus371 | 34 | 874.0659 | 0.2146 | 0.3975 | 3 | 0.35008 | 0.6790 |
| Genus503 | 16 | 187.2604 | 0.1102 | 0.4833 | 3 | -1.12420 | 0.5556 |
| Genus522 | 21 | 132.8431 | 0.1634 | 0.6095 | 3 | 0.27505 | 0.4694 |
| Genus716 | 27 | 1327.3440 | 0.1465 | 0.3077 | 3 | -0.64341 | 0.6331 |
| Genus29 | 29 | 1457.9330 | 0.1794 | 0.3744 | 3 | -0.02986 | 0.2099 |
| Genus35 | 11 | 102.1725 | 0.0245 | 0.5455 | 3 | -0.15316 | 0.6875 |
| Genus88 | 11 | 57.5358 | 0.0449 | 0.5636 | 3 | -1.16567 | 0.0000 |
| Genus80 | 24 | 827.4792 | 0.1644 | 0.4964 | 3 | 0.58908 | 0.5014 |
| Genus102 | 15 | 540.7374 | 0.0545 | 0.3714 | 3 | 0.53971 | 0.7153 |
| Genus105 | 1 | 0.0000 | 0.0026 | NA | 3 | -0.95827 | 0.7500 |
| Genus198 | 17 | 1188.4627 | 0.0169 | 0.2941 | 3 | 0.12487 | 0.6471 |
| Genus114 | 20 | 428.8255 | 0.1150 | 0.4105 | 3 | 1.42762 | 0.4675 |
| Genus213 | 13 | 321.9623 | 0.0294 | 0.5641 | 3 | 1.03935 | 0.5788 |
| Genus211 | 10 | 258.8336 | 0.0543 | 0.4667 | 3 | -0.16262 | 0.3846 |
| Genus212 | 35 | 1175.0609 | 0.2281 | 0.3580 | 3 | -1.26775 | 0.0000 |
| Genus215 | 18 | 952.9880 | 0.0345 | 0.3399 | 3 | 0.12487 | 0.4097 |
| Genus216 | 5 | 60.7860 | 0.0241 | 0.6000 | 3 | -1.00400 | 0.6000 |
| Genus218 | 29 | 552.6796 | 0.1759 | 0.4335 | 3 | -0.18460 | 0.2344 |
| Genus200 | 7 | 60.4429 | 0.0420 | 0.5714 | 3 | 1.05329 | 0.2994 |
| Genus235 | 25 | 532.9186 | 0.1615 | 0.4233 | 3 | -0.30124 | 0.6000 |
| Genus331 | 21 | 336.4446 | 0.1425 | 0.5190 | 3 | 0.43435 | 0.3622 |
| Genus421 | 24 | 1174.0808 | 0.1345 | 0.3225 | 3 | -0.15316 | 0.5556 |
| Genus360 | 6 | 30.6585 | 0.0343 | 0.6000 | 3 | 0.00000 | 0.0000 |
| Genus380 | 14 | 1146.2377 | 0.0726 | 0.3956 | 3 | 2.60191 | 0.0580 |
| Genus344 | 9 | 7.3270 | 0.0492 | 0.8056 | 3 | -1.15965 | 0.0000 |
| Genus435 | 31 | 743.0164 | 0.2180 | 0.4452 | 3 | 1.13947 | 0.4545 |
| Genus412 | 4 | 39.1349 | 0.0231 | 0.3333 | 3 | 1.05329 | 0.2932 |
| Genus296 | 8 | 151.6513 | 0.0092 | 0.4286 | 3 | 1.52014 | 0.0784 |
| Genus474 | 5 | 2.2563 | 0.0374 | 0.6000 | 3 | 1.76053 | 0.2862 |
| Genus513 | 14 | 191.3747 | 0.1069 | 0.5714 | 3 | -0.52321 | 0.2344 |
| Genus505 | 24 | 1095.5319 | 0.1500 | 0.4130 | 3 | 1.35657 | 0.3056 |
| Genus511 | 12 | 44.9247 | 0.0749 | 0.6515 | 3 | 1.15955 | 0.2274 |
| Genus554 | 25 | 151.7651 | 0.1893 | 0.5433 | 3 | -1.00400 | 0.6000 |
| Genus591 | 14 | 32.5765 | 0.1088 | 0.6923 | 3 | 0.00000 | 0.0000 |
| Genus552 | 15 | 500.8680 | 0.0819 | 0.4190 | 3 | -0.95827 | 0.5556 |
| Genus625 | 17 | 409.3316 | 0.0928 | 0.4412 | 3 | -0.04242 | 0.1597 |
| Genus443 | 3 | 11.4521 | 0.0008 | 0.3333 | 3 | -1.26775 | 0.0000 |
| Genus637 | 21 | 1458.7151 | 0.1055 | 0.3190 | 3 | 0.35008 | 0.6389 |
| Genus712 | 19 | 364.1292 | 0.1123 | 0.4327 | 3 | 1.22474 | 0.4375 |
| Genus195 | 1 | 0.0000 | 0.0027 | NA | 3 | 0.85133 | 0.5537 |
| Genus292 | 1 | 0.0000 | 0.0008 | NA | 3 | 0.07777 | 0.4740 |
| Genus407 | 1 | 0.0000 | 0.0017 | NA | 3 | -1.12420 | 0.5556 |
| Genus598 | 13 | 27.2595 | 0.0962 | 0.6282 | 3 | -0.87753 | 0.7778 |
| Genus602 | 1 | 0.0000 | 0.0015 | NA | 3 | 0.43837 | 0.2932 |
| Genus651 | 3 | 9.1101 | 0.0020 | 0.6667 | 3 | 0.43837 | 0.5748 |
| Genus703 | 18 | 639.1459 | 0.0824 | 0.4314 | 3 | 0.00000 | 0.5556 |
| Genus733 | 1 | 0.0000 | 0.0045 | NA | 3 | -0.16262 | 0.1736 |
| Genus543 | 3 | 3.6765 | 0.0102 | 0.3333 | 3 | 0.12487 | 0.4097 |
| Genus353 | 1 | 0.0000 | 0.0001 | NA | 4 | 0.27505 | 0.3056 |
| Genus441 | 6 | 236.0357 | 0.0007 | 0.6000 | 4 | -0.65640 | 0.7500 |
| Genus343 | 9 | 1469.1181 | 0.0233 | 0.2500 | 4 | 1.67223 | 0.0975 |
| Genus526 | 3 | 860.2618 | 0.0011 | 0.0000 | 4 | 1.22474 | 0.4375 |
| Genus176 | 6 | 51.1481 | 0.0014 | 0.6667 | 4 | -0.65640 | 0.7500 |
| Genus120 | 4 | 481.4016 | 0.0003 | 0.1667 | 4 | -1.36459 | 0.0000 |
| Genus190 | 2 | 200.9058 | 0.0012 | 0.0000 | 4 | 1.05329 | 0.2932 |
| Genus20 | 1 | 0.0000 | 0.0000 | NA | 4 | -1.36459 | 0.0000 |
| Genus189 | 8 | 366.2954 | 0.0071 | 0.5000 | 4 | -0.57735 | 0.0000 |
| Genus392 | 2 | 233.0000 | 0.0001 | 0.0000 | 4 | 1.42762 | 0.3223 |
| Genus369 | 8 | 182.8603 | 0.0033 | 0.4643 | 4 | -0.01310 | 0.5918 |
| Genus454 | 10 | 938.5377 | 0.0022 | 0.2444 | 4 | -1.16567 | 0.0000 |
| Genus455 | 6 | 55.0517 | 0.0007 | 0.6667 | 4 | -1.12420 | 0.6875 |
| Genus351 | 3 | 297.7410 | 0.0001 | 0.0000 | 4 | 0.00000 | 0.0000 |
| Genus568 | 2 | 0.0000 | 0.0000 | 1.0000 | 4 | -0.95027 | 0.0000 |
| Genus525 | 6 | 206.7497 | 0.0138 | 0.5333 | 4 | -0.80354 | 0.6000 |
| Genus460 | 4 | 376.6439 | 0.0002 | 0.1667 | 4 | 0.91915 | 0.1791 |
| Genus646 | 10 | 338.4606 | 0.0297 | 0.4000 | 4 | 0.06350 | 0.7143 |
| Genus397 | 1 | 0.0000 | 0.0000 | NA | 4 | 0.35008 | 0.6500 |
| Genus592 | 2 | 0.0000 | 0.0000 | 1.0000 | 4 | -1.16567 | 0.0000 |
| Genus647 | 3 | 54.2923 | 0.0010 | 0.3333 | 4 | -0.49407 | 0.4694 |
| Genus668 | 2 | 124.0922 | 0.0000 | 0.0000 | 4 | -0.64880 | 0.3600 |
| Genus726 | 1 | 0.0000 | 0.0000 | NA | 4 | -1.16567 | 0.0000 |
| Genus133 | 4 | 404.1295 | 0.0000 | 0.1667 | 5 | 0.27961 | 0.5465 |
| Genus152 | 3 | 710.3215 | 0.0002 | 0.3333 | 5 | 1.52043 | 0.4375 |
| Genus126 | 2 | 767.0678 | 0.0004 | 0.0000 | 5 | 2.75538 | 0.2433 |
| Genus174 | 4 | 463.9546 | 0.0000 | 0.1667 | 5 | 1.35657 | 0.5781 |
| Genus139 | 3 | 185.8578 | 0.0000 | 0.3333 | 5 | 1.67223 | 0.3006 |
| Genus346 | 4 | 1022.2963 | 0.0072 | 0.0000 | 5 | 0.79896 | 0.2608 |
| Genus362 | 3 | 615.6091 | 0.0004 | 0.0000 | 5 | -0.40301 | 0.2099 |
| Genus430 | 4 | 604.4322 | 0.0000 | 0.1667 | 5 | -0.95827 | 0.6875 |
| Genus160 | 2 | 555.1972 | 0.0000 | 0.0000 | 5 | -0.61237 | 0.0000 |
| Genus168 | 1 | 0.0000 | 0.0000 | NA | 5 | -0.95827 | 0.5556 |
| Genus188 | 1 | 0.0000 | 0.0000 | NA | 5 | -0.30124 | 0.4375 |
| Genus368 | 2 | 0.0000 | 0.0000 | 1.0000 | 5 | -0.95827 | 0.6875 |
| Genus476 | 1 | 0.0000 | 0.0000 | NA | 5 | -0.80354 | 0.6000 |
| Genus47 | 26 | 653.5957 | 0.1516 | 0.3815 | 6 | 0.00000 | 0.0000 |
| Genus142 | 12 | 759.1264 | 0.0235 | 0.2273 | 6 | -0.95027 | 0.0000 |
| Genus178 | 4 | 13.2947 | 0.0072 | 0.6667 | 6 | -0.61237 | 0.7500 |
| Genus254 | 14 | 376.3679 | 0.0597 | 0.3846 | 6 | -1.26775 | 0.7500 |
| Genus256 | 4 | 4.8078 | 0.0143 | 0.5000 | 6 | -0.80354 | 0.4375 |
| Genus348 | 5 | 17.2539 | 0.0188 | 0.7000 | 6 | 0.07777 | 0.5399 |
| Genus473 | 13 | 199.0152 | 0.0431 | 0.3846 | 6 | -0.33933 | 0.5500 |
| Genus453 | 4 | 4.9732 | 0.0256 | 0.6667 | 6 | -0.15316 | 0.6875 |
| Genus534 | 4 | 5.8139 | 0.0085 | 0.5000 | 6 | -0.87753 | 0.7500 |
| Genus559 | 10 | 123.8550 | 0.0382 | 0.3778 | 6 | 0.07777 | 0.4180 |
| Genus542 | 7 | 78.5477 | 0.0439 | 0.5238 | 6 | -0.16262 | 0.4439 |
| Genus466 | 1 | 0.0000 | 0.0008 | NA | 6 | 1.01593 | 0.5900 |
| Genus582 | 7 | 57.2632 | 0.0216 | 0.5714 | 6 | 1.15470 | 0.7778 |
| Genus687 | 4 | 1.4636 | 0.0259 | 0.8333 | 6 | 1.82697 | 0.0930 |
| Genus705 | 7 | 276.3024 | 0.0156 | 0.3810 | 6 | -0.16262 | 0.1736 |
| Genus450 | 1 | 0.0000 | 0.0000 | NA | 7 | -1.36515 | 0.0000 |
| Genus566 | 5 | 696.0000 | 0.0000 | 0.1000 | 7 | 0.53971 | 0.6672 |
| Genus607 | 1 | 0.0000 | 0.0000 | NA | 7 | 0.06350 | 0.7551 |
| Genus23 | 2 | 410.9663 | 0.0001 | 0.0000 | 7 | -0.04242 | 0.1597 |
| Genus414 | 4 | 1478.4395 | 0.0172 | 0.3333 | 7 | -0.18460 | 0.2344 |
| Genus479 | 1 | 0.0000 | 0.0000 | NA | 7 | 1.67223 | 0.5193 |
| Genus361 | 1 | 0.0000 | 0.0000 | NA | 7 | -0.95827 | 0.6875 |
| Genus364 | 1 | 0.0000 | 0.0000 | NA | 7 | -0.41272 | 0.6875 |
| Genus587 | 4 | 1675.9269 | 0.0009 | 0.1667 | 7 | -1.26775 | 0.0000 |
| Genus686 | 4 | 465.0000 | 0.0000 | 0.1667 | 7 | -1.15965 | 0.0000 |
| Genus30 | 2 | 17.5573 | 0.0024 | 0.0000 | 8 | 0.27961 | 0.1736 |
| Genus84 | 3 | 57.4198 | 0.0116 | 0.3333 | 8 | -0.33933 | 0.5309 |
| Genus310 | 1 | 0.0000 | 0.0000 | NA | 9 | -0.88894 | 0.6327 |
| Genus482 | 1 | 0.0000 | 0.0000 | NA | 9 | -1.12420 | 0.5556 |
| Genus688 | 3 | 465.0000 | 0.0007 | 0.0000 | 9 | -1.36459 | 0.0000 |
| Genus180 | 1 | 0.0000 | 0.0000 | NA | 10 | -0.33933 | 0.5062 |
| Genus484 | 1 | 0.0000 | 0.0000 | NA | 10 | 2.24132 | 0.1699 |
| Genus389 | 1 | 0.0000 | 0.0000 | NA | 11 | -0.18460 | 0.2344 |
| Genus480 | 1 | 0.0000 | 0.0000 | NA | 11 | -0.65640 | 0.7500 |
| Genus370 | 1 | 0.0000 | 0.0000 | NA | 12 | -1.00400 | 0.6000 |
| Genus449 | 1 | 0.0000 | 0.0000 | NA | 12 | -1.11301 | 0.7500 |
| Genus116 | 1 | 0.0000 | 0.0000 | NA | 13 | -0.58938 | 0.5556 |
| Genus130 | 1 | 0.0000 | 0.0000 | NA | 13 | -1.36459 | 0.0000 |

**References:**

1. Mizuno CM, Rodriguez-Valera F, Kimes NE, Ghai R. 2013. Expanding the Marine Virosphere Using Metagenomics. PLos Genet 9.
